# Sharkskin-Inspired Magnetoactive Reconfigurable Acoustic Metamaterials

**DOI:** 10.34133/2020/4825185

**Published:** 2020-02-05

**Authors:** Kyung Hoon Lee, Kunhao Yu, Hasan Al Ba'ba'a, An Xin, Zhangzhengrong Feng, Qiming Wang

**Affiliations:** Sonny Astani Department of Civil and Environmental Engineering, University of Southern California, Los Angeles, CA 90089, USA

## Abstract

Most of the existing acoustic metamaterials rely on architected structures with fixed configurations, and thus, their properties cannot be modulated once the structures are fabricated. Emerging active acoustic metamaterials highlight a promising opportunity to on-demand switch property states; however, they typically require tethered loads, such as mechanical compression or pneumatic actuation. Using untethered physical stimuli to actively switch property states of acoustic metamaterials remains largely unexplored. Here, inspired by the sharkskin denticles, we present a class of active acoustic metamaterials whose configurations can be on-demand switched via untethered magnetic fields, thus enabling active switching of acoustic transmission, wave guiding, logic operation, and reciprocity. The key mechanism relies on magnetically deformable Mie resonator pillar (MRP) arrays that can be tuned between vertical and bent states corresponding to the acoustic forbidding and conducting, respectively. The MRPs are made of a magnetoactive elastomer and feature wavy air channels to enable an artificial Mie resonance within a designed frequency regime. The Mie resonance induces an acoustic bandgap, which is closed when pillars are selectively bent by a sufficiently large magnetic field. These magnetoactive MRPs are further harnessed to design stimuli-controlled reconfigurable acoustic switches, logic gates, and diodes. Capable of creating the first generation of untethered-stimuli-induced active acoustic metadevices, the present paradigm may find broad engineering applications, ranging from noise control and audio modulation to sonic camouflage.

## 1. Introduction

Acoustic metamaterials with tailored architectures exhibit unconventional capability in controlling acoustic waves [1–5] and have enabled a wide range of previously unachievable applications, such as superlensing [6–12], cloaking [13–15], logic operation [16–20], nonreciprocal propagation [21–25], topological insulation [20, 26–29], and wave guiding [30, 31]. Despite the diverse applications, most of the existing paradigms rely on architected structures with fixed configurations, and thus, their properties cannot be modulated once the structures are fabricated [1–5]. Emerging active acoustic metamaterials highlight a special opportunity to enable on-demand property switching of the fabricated acoustic metamaterials [32, 33]. However, the existing active metamaterials typically require tethered loads, such as mechanical compression or pneumatic actuation [20, 30, 31, 34]. Compared to tethered loads, untethered physical stimuli (such as electromagnetic field, light, and temperature) are more appealing owing to their special advantages including noncontact with the structures, nonlocal modulation, and rapid switching [35, 36]. However, using untethered physical stimuli to on-demand switch property states of acoustic metamaterials remains largely unexplored [35, 36]. Although there are recent reports on using the electromagnetic fields to modulate the effective constitutive parameters of acoustic metamaterials [35, 36], using untethered stimuli to enable reconfigurable acoustic devices such as waveguide, logic gates, or nonreciprocal diodes remains elusive.

Here, we report a class of active acoustic metamaterials whose configurations can be on-demand switched via untethered magnetic fields, thus enabling active switching of acoustic transmission, wave guiding, logic operation, and reciprocity. The key mechanism relies on magnetically deformable Mie resonator pillar (MRP) arrays that can be tuned between vertical and bent states corresponding to the acoustic forbidding and conducting, respectively. The MRPs are made of an iron-filled magnetoactive elastomer and feature wavy air channels to enable an artificial Mie resonance within a designed frequency regime [37–39]. The Mie resonance induces an acoustic bandgap, which is closed when pillars are selectively bent by a sufficiently large magnetic field (e.g., 0.13 T). These magnetoactive MRPs are further harnessed to design stimuli-controlled reconfigurable acoustic switches (i.e., shifting between different propagation pathways), reconfigurable acoustic logic gates (i.e., switching among NOT, AND, and OR gates), and reconfigurable acoustic diodes (i.e., switching between the nonreciprocal diode and reciprocal conductor). Integrating stimuli-responsive smart materials and Mie resonances, the present paradigm highlights a unique and promising avenue for acoustic metamaterials that can reversibly, repeatedly, and on-demand switch acoustic propagation, logic operation, and reciprocity via untethered physical stimuli.

## 2. Sharkskin-Inspired Design Principle

**Figure 1:**
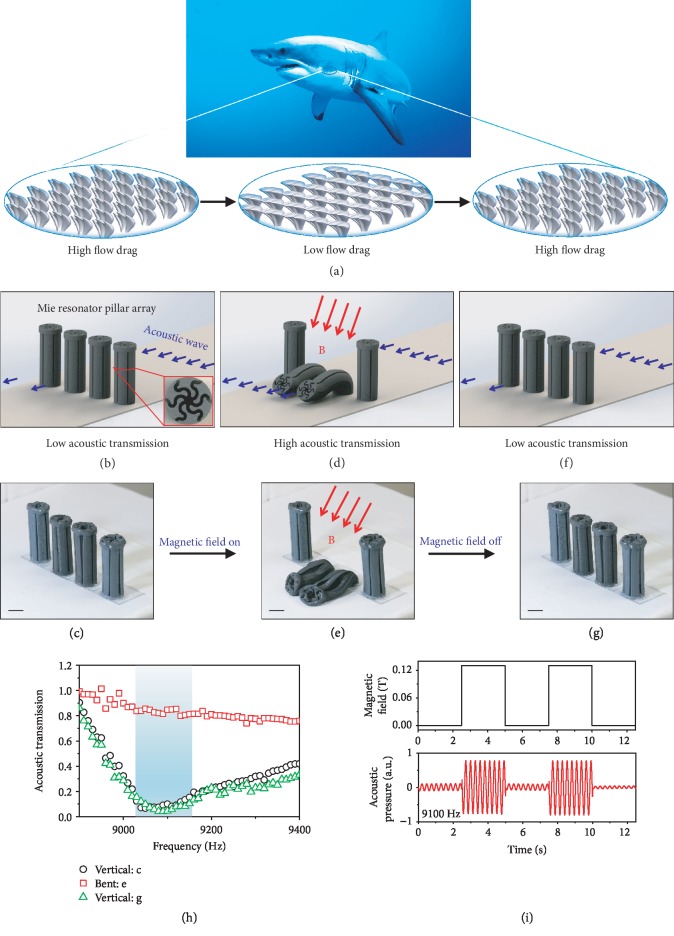
Sharkskin-inspired design principle of the magnetoactive reconfigurable acoustic metamaterials. (a) Schematics to show the sharkskin denticles for switchable flow drag via reversibly tilting the denticles. (b, c) Schematic and sample of the vertical Mie resonator pillar array. Inset in (b) shows the cross-section of the pillar. (d, e) Schematic and sample of the pillar array with two pillars bent via a magnetic field. The red arrows indicate the direction of the magnetic field. The selective actuation of the central two pillars is because of their higher iron volume fraction (18.48% by weight, Fig. [Supplementary-material supplementary-material-1]). (f, g) Schematic and sample of the pillar array when the magnetic field is turned off. (h) The acoustic transmissions of samples corresponding to (c), (e), and (g) in functions of the frequency. (i) The applied magnetic field intensity and the corresponding acoustic pressure at 9100 Hz within two switching cycles. The scale bars in (c), (e), and (g) represent 1 cm. The photo credit of the shark in (a) comes from Braulio Lopez Gonzalez Jr.

The design principle of magnetoactive reconfigurable acoustic metamaterials is inspired by the sharkskin [40–42]. Fast-swimming sharks feature skin denticles that are shaped like “V” trenches and aligned in the direction of fluid flow (Figure 1(a)). It has been proven that the aligned skin denticles can significantly reduce the flow drag because the V-shaped trenches can guide a turbulent flow to become a laminate flow [43]. It has been further discovered that tilting the skin denticles by a small angle can drastically increase the flow drag [44]. Sharks can smartly switch the skin flow drag by reversibly tilting the skin denticles (Figure 1(a)) [41, 45].

Inspired by this natural paradigm in tuning the flow drag, we here propose a class of magnetoactive MRP arrays that can smartly switch the acoustic transmission by reversibly bending MRPs via a remotely controlled magnetic field (Figure 1(b)–(g)). Each MRP (diameter *D* = 1.5 cm and height *H* = 4.25 cm) has six-section wavy air channels that enable an artificial Mie resonance within a designed frequency regime [37–39]. When the pillar spacing *L* is relatively small (e.g., *L*/*D* = 1.5), the Mie resonances around adjacent MRPs are coupled to form an acoustic barrier to enable a low acoustic transmission within 9040-9140 Hz (<0.2, Figure 1(b), (c), and (h)). Besides, the pillars are made of a magnetoactive elastomer reinforced by ferromagnetic iron nanoparticles (see Materials and Methods and Fig. [Supplementary-material supplementary-material-1]), and thus, they can be selectively bent via a sufficiently large magnetic field (e.g., 0.13 T) to open a large pillar-to-pillar spacing for the remaining vertical pillars, leading to a high acoustic transmission within 9040-9140 Hz (>0.8, Figure 1(d), (e), and (h)). When the remotely controlled magnetic field is reduced or turned off, the bent MRPs return to the vertical state, and thus, the acoustic transmission turns back to the low level within 9040-9140 Hz (<0.2, Figure 1(f)–(h)). Since the pillar deformation is fully elastic, the pillar bending is rapid, reversible, and repeatable, enabling on-demand switching of the acoustic transmission by tuning the applied magnetic field over multiple cycles (Figure 1(i), [Supplementary-material supplementary-material-1] with cycle period 5 s, and [Supplementary-material supplementary-material-1] with cycle period 1 s).

## 3. Mechanism of the MRP Array

**Figure 2:**
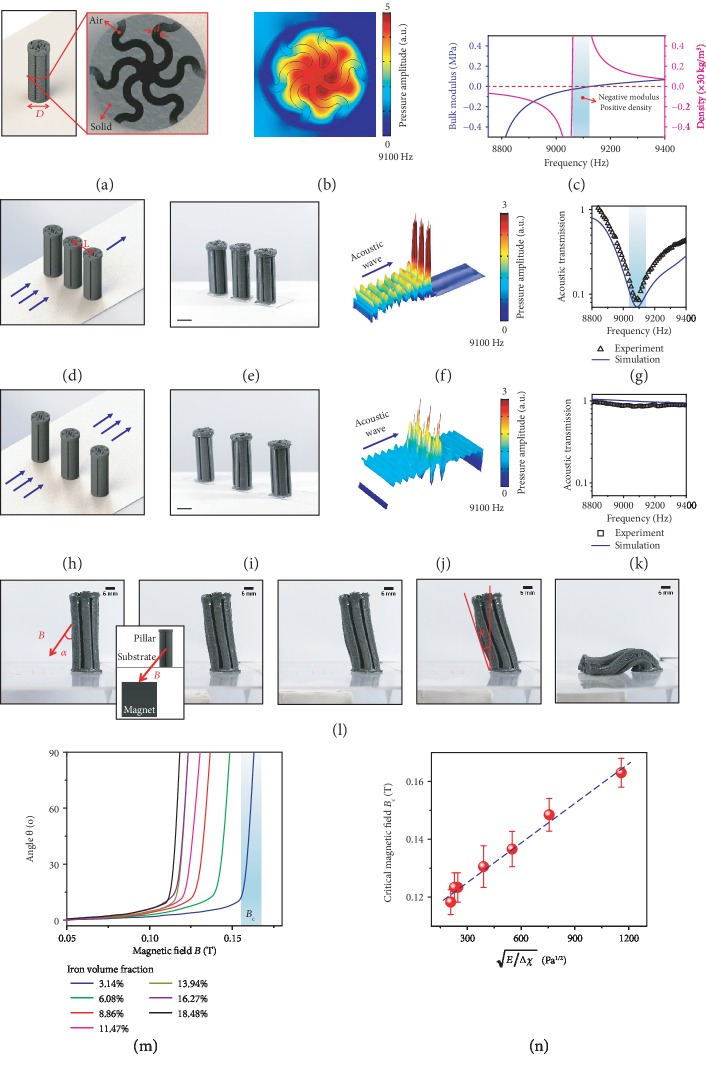
Mechanism of MRPs and their magnetically induced buckling. (a) Schematics to show the geometry of a Mie resonator pillar. (b) Numerically simulated acoustic pressure around an MRP at 9100 Hz. (c) Numerically calculated effective bulk modulus and density of an MRP in functions of the acoustic frequency. The shaded area indicates a regime with negative modulus and positive density. (d, e) Schematic and sample for an MRP array with a small spacing (*L*/*D* = 1.5). (f) Numerically simulated pressure of an acoustic wave moving through the MRP array with a small spacing (*L*/*D* = 1.5). (g) The experimentally measured and numerically simulated acoustic transmission of the MRP array with a small space in functions of the frequency. The shadowed area indicates the Mie resonance frequency regime. (h, i) Schematic and sample for an MRP array with a large spacing (*L*/*D* = 2.5). (j) Numerically simulated pressure of an acoustic wave moving through the MRP array with a large spacing (*L*/*D* = 2.5). (k) The experimentally measured and numerically simulated acoustic transmission of the MRP array with a large spacing in functions of the frequency. (l) Image sequence to show the magnetically induced bending of an MRP with increasing magnetic fields. The inset shows a schematic for the application of the magnetic field to the MRP. (m) Bending angles of MRPs with various volume fractions of the iron particle in functions of the magnetic field. The shaded area indicates the critical magnetic field *B*_c_. (n) The experimentally measured critical magnetic fields for various volume fractions of the iron particle in a function of $\sqrt{E/\Delta \chi }$, where *E* is Young's modulus of the elastomer and Δ*χ* is the effective magnetic susceptibility difference. The error bars indicate the variation of the magnetic field within the shaded area in (m). Scale bars in (e) and (i) denote 1 cm.

Next, we analyze the mechanism of the MRP array. An MRP features six-section wavy air channels with the relative width as *d*/*D* = 0.07 (Figure 2(a)). The propagation length of the acoustic wave within the air channel is longer than that in the solid medium. Effectively, the acoustic energy within a certain frequency regime is trapped within the air channels [37]. This point can be verified by a numerical simulation which shows that the acoustic energy is concentrated within the MRP center region around 9100 Hz (Figure 2(b) and [Supplementary-material supplementary-material-1]). This phenomenon can be explained as an artificial Mie resonance within the MRP structure [37–39, 46, 47]. From the perspective of an effective medium, this Mie resonance is corresponding to negative effective bulk modulus and positive effective density within frequency 9050-9120 Hz (Figure 2(c) and [Supplementary-material supplementary-material-1]). This frequency regime (9050-9120 Hz) is considered as the Mie resonance frequency. This frequency regime is consistent with the numerically simulated bandgap (Fig. [Supplementary-material supplementary-material-1]). To verify this Mie resonance frequency, we further carry out analytical modeling of the MRP using an equivalent multiphase composite model (Supplementary Information, Fig. [Supplementary-material supplementary-material-1]) [37, 48, 49]. The analytical model shows that the Mie resonance frequency of the MRP with diameter 1.5 cm and 64% solid volume fraction is ~8.9 kHz ([Supplementary-material supplementary-material-1]), which is relatively close to the numerical simulations (Figure 2(c)).

The MRPs can be harnessed to shield the acoustic wave if multiple MRPs are arranged in an array (Figure 2(d) and (e)). When the pillar spacing *L* is relatively small (e.g., *L*/*D* = 1.5), the Mie resonances in adjacent pillars are coupled to construct an acoustic shielding layer to block the acoustic transmission around the Mie resonance frequency regime (Figure 2(d) and (e)). This acoustic forbidding behavior at 9100 Hz is visualized by a numerical simulation shown in Figure 2(f) (Fig. [Supplementary-material supplementary-material-1]): the acoustic pressure amplitude drastically decreases to a very small value (<0.05) behind the MRP array. This behavior is validated by the experimentally measured acoustic transmission through the MRP array (Figure 2(g)): the acoustic transmission becomes lower than 0.15 within 9050-9120 Hz. The experimentally measured acoustic transmissions roughly agree with the numerical simulations over 8800-9400 Hz (Figure 2(g)). When the pillar spacing is relatively large (e.g., *L*/*D* = 2.5), the acoustic wave can escape between MRPs to enable a relatively high acoustic transmission (>0.8, Figure 2(h)–(k) and [Supplementary-material supplementary-material-1]). To verify this phenomenon, we study a control pillar array with solid pillars without air channels (*D* = 1.5 cm and *L*/*D* = 1.5) (Fig. [Supplementary-material supplementary-material-1]). Both experiments and simulations show the acoustic transmissions over 8800-9400 Hz are above 0.8 (Fig. [Supplementary-material supplementary-material-1]), implying that the air channels within the MRPs really play an important role to enable the low acoustic transmission around 9100 Hz. To further verify the phenomenon, we gradually vary the pillar spacing and numerically simulate the acoustic transmission through the MRP array. We find the acoustic transmission increases with increasing pillar spacing within 9050-9150 Hz (Fig. [Supplementary-material supplementary-material-1]).

In this work, the enlargement of the pillar spacing is enabled by the bending of selected pillars in the pillar array (Figure 1(d) and (e)). Once selected pillars are bent, the remaining vertical pillars construct a pillar array with a larger spacing (i.e., *L*/*D* = 4.6) that leads to high acoustic transmission.

## 4. Magnetically Induced Bending of MRPs

To achieve precise control of the magnetically induced bending of MRPs, we need to understand the underlining mechanics. We first carry out experiments on the magnetically induced bending of a bottom-fixed MRP under a magnetic field *B* with a tilted angle *α* (Figures 2(l) and [Supplementary-material supplementary-material-1]). With increasing magnetic field *B*, the pillar bends by an angle *θ* (Figure 2(l)). When the angle *θ* reaches 15-18°, further increasing *B* will suddenly bend and pinch the pillar onto the substrate. This can be understood as a critical condition for the magnetically induced buckling of the MRP. To quantify this phenomenon, we plot the bending angle *θ* in a function of the applied magnetic field (Figure 2(m)): after the critical magnetic field *B*_c_, the bending angle *θ* abruptly reaches 90°.

We then develop an analytical model to understand the magnetically induced buckling of the MRP. Using a similar analysis in the previously reported study on magnetically induced bulking of tilted beams (Supplementary Information, Fig. [Supplementary-material supplementary-material-1]) [35], we express the critical magnetic field of the buckling as

(1) $$\begin{array}{c} B_{\text{c}}=\beta \left(\alpha ,\frac{H}{D}\right)\sqrt{\frac{\mu_{0}E}{\Delta \chi }\left(\frac{I}{AH^{2}}\right)}, \end{array}$$

where *β* is a dimensionless parameter dependent on the magnetic field angle *α* and the aspect ratio of the MRP *H*/*D*, *μ*_0_ is the magnetic permittivity of the vacuum, *E* is Young's modulus of the elastomer, *I* is the second moment of area, *A* is the cross-section area of the solid part, and *H* is the pillar length. Δ*χ* is the effective magnetic susceptibility difference between the axial and orthogonal directions and can be estimated as Δ*χ* ≈ *χ* − *χ*/(1 + *χ*/2), where *χ* is the magnetic susceptibility of the elastomer. To validate Equation (1), we maintain the geometry of the pillar and vary the volume fraction of the ferromagnetic iron particle within the magnetoactive elastomer. We find that the critical magnetic field for the magnetically induced buckling decreases as the volume fraction of the iron particle increases from 3.14% to 18.48% (Figure 2(m)). According to Equation (1), when the geometry parameters and the magnetic field angle maintain constant, the critical magnetic field *B*_c_ should scale with $\sqrt{E/\Delta \chi }$. As shown in Figure 2(n), this scaling law agrees with the experimental results for MRPs with various volume fractions of the iron particle (parameters are listed in [Supplementary-material supplementary-material-1]).

Note that to enable the magnetic buckling of selected pillars shown in Figure 1(d) and (e), we use pillars with higher iron volume fraction at selected locations. For example, the central two MRPs have an iron volume fraction of 18.48% but the other two have an iron volume fraction of 3.14% in Figure 1(e). When a sufficiently large magnetic field (0.13 T) is applied, only the central two MRPs buckle. It should also be noted that the inhomogeneity of the iron volume fraction among four MRPs does not change the key physics of the Mie resonance shown in Figure 2(a)–(k). The numerical simulations of MRP array with inhomogeneous iron volume fractions display a low acoustic transmission within 9050-9150 Hz (Fig. [Supplementary-material supplementary-material-1]), similar to that of the MRP array with homogeneous iron volume fraction (Figure 2(g)). The numerical results of the MRP array with inhomogeneous iron volume fractions also agree with the corresponding experimental results in Figure 1(h) (Fig. [Supplementary-material supplementary-material-1]).

## 5. Magnetoactive Acoustic Double-Throw Switch

**Figure 3:**
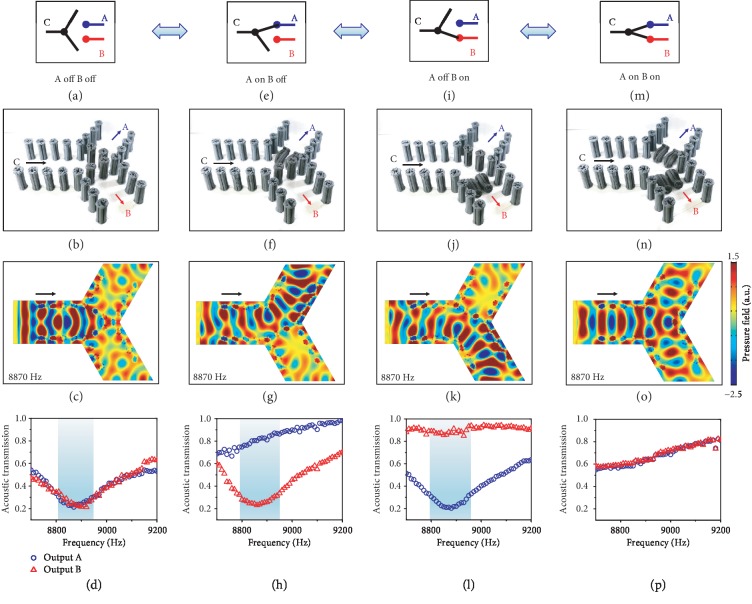
Magnetoactive acoustic double-throw switch. Schematics (a, e, i, and m), samples (b, f, j, and n), numerical simulations (c, g, k, and o), and experimentally measured transmission-frequency results (d, h, l, and p) of four function states of a magnetoactive acoustic double-throw switch: “A off B off” (a–d), “A on B off” (e–h), “A off B on” (i–l), and “A on B on” (m–p). Note that the selective actuation of the central four pillars is because of their higher iron volume fraction (18.48% by weight). The bent pillars are removed in the simulations of (g), (k), and (o).

Next, we harness MRPs to design a magnetoactive acoustic double-throw switch (Figure 3). We first use MRPs to design a three-branch channel with two outputs (A and B) and one input (C) (Figure 3(a) and (b)). The channel wall is composed of MRP arrays with a low iron volume fraction (3.14%) and a small pillar spacing (*L*/*D* = 1.5) to prevent the acoustic leaking within a designed frequency regime. Two sets of MRPs with a high iron volume fraction (18.48%) and a small pillar spacing (*L*/*D* = 1.5) are located on the pathways of C-to-A and C-to-B. At the as-fabricated state, the acoustic transmissions of C-to-A and C-to-B are expected to be blocked by the MRPs around the Mie resonance frequency. This point can be first validated by numerical simulations that show the drastically reduced acoustic pressure at channels A and B (Figures 3(c) and [Supplementary-material supplementary-material-1]). Experiments show that the acoustic transmissions of C-to-A and C-to-B are both below 0.25 within 8800-8950 Hz (Figure 3(d)). We denote the as-fabricated state as “A off B off” state (Figure 3(a)–(d)). Note that this Mie resonance frequency region (8800-8950 Hz) is slightly different from that shown in Figures 1 and 2 (9050-9120 Hz). This difference can be explained by the geometrical inconsistency among different fabricated MRPs: numerical simulations show that the Mie resonance frequency varies from 8620 Hz to 9300 Hz by varying the pillar diameter by 7% (Fig. [Supplementary-material supplementary-material-1]). This effect of pillar diameter variation can also be verified by the analytical modeling of an MRP which shows that the Mie resonance frequency changes from 9480 Hz to 8300 Hz by varying the pillar diameter from 1.4 to 1.6 cm (Fig. [Supplementary-material supplementary-material-1], Supplementary Information).

To switch the “A off B off” state to “A on B off” state, we use a magnetic field (0.13 T) to bend the MRPs on the pathway of C-to-A (Figure 3(e) and (f)). Numerical simulations show that the acoustic pressure at channel A increases drastically after bending the MRPs (Figure 3(g) and [Supplementary-material supplementary-material-1]). Experiments show that the transmission of C-to-A increases to ~0.8 within 8800-8950 Hz, while the transmission of C-to-B remains below 0.25 (Figure 3(h)). Similarly, when the MRPs on the pathway of C-to-B are bent by a magnetic field, the switch transforms to “A off B on” state (Figure 3(i)–(l) and [Supplementary-material supplementary-material-1]). When the MRPs on the pathway of C-to-A and C-to-B are all bent by respective magnetic fields, the switch transforms to the state of “A on B on” (Figure 3(m)–(p) and [Supplementary-material supplementary-material-1]). The above four states of the double-throw switch can be on-demand and reversibly modulated via the remotely controlled magnetic fields.

## 6. Magnetoactive Reconfigurable Acoustic Logic Gate

**Figure 4:**
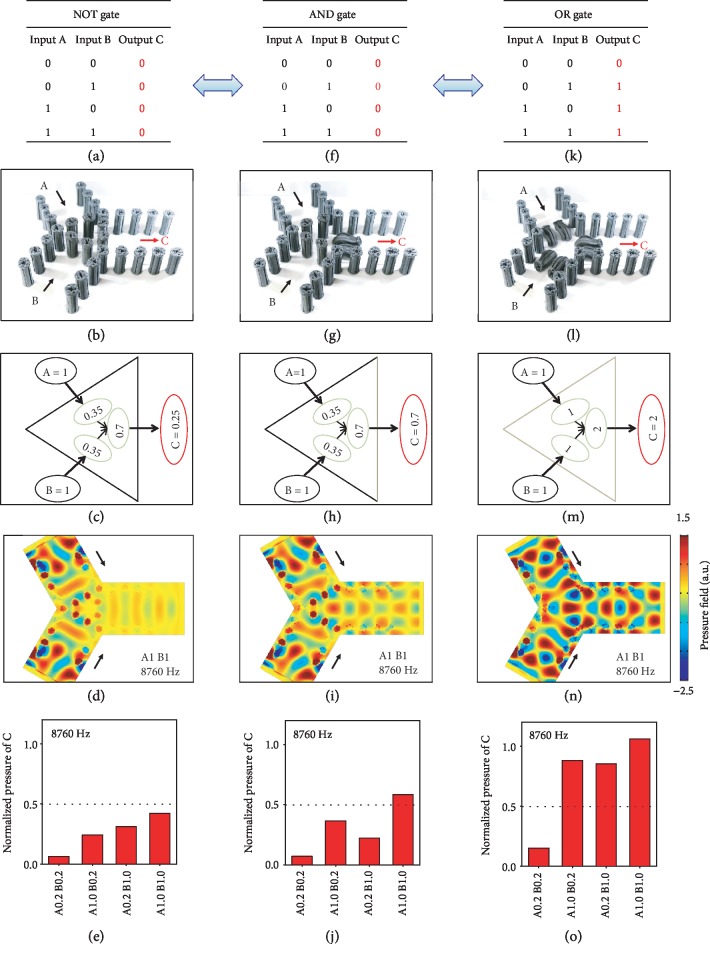
Magnetoactive reconfigurable acoustic logic gates. Operation schemes (a, f, and k), samples (b, g, and l), simplified operation schemes with normalized acoustic pressure (c, h, and m), numerical simulations for the case of “A1B1” at 8760 Hz (d, i, and n), and measured normalized pressures of output C (e, j, and o) of NOT gate (a–e), AND gate (f–j), and OR gate (k–o), respectively. “A#B#” in (d), (h), and (l) indicates the normalized pressures in inputs A and B, respectively. The normalized pressure values shown in (c), (e), (h), (j), (m), and (o) are calculated by normalizing the pressure amplitude values by the acoustic pressure generated by the speaker with the power input of 1 V at 8760 Hz. Note that the selective actuation of the central six pillars is because of their higher iron volume fraction (18.48% by weight). The bent pillars are removed in the simulations of (i) and (n).

Next, we harness MRPs to design magnetoactive reconfigurable acoustic logic gates (Figure 4). Existing acoustic logic gates primarily rely on designed acoustic metastructures with fixed geometries [16–19], and very few of them can switch logic operators with tethered interventions [20]. Reconfigurable acoustic logic gates that can on-demand switch operators by untethered stimuli have not been explored. Here, we demonstrate a magnetoactive reconfigurable acoustic logic gate with a three-branch channel slightly modified from the acoustic switch shown in Figure 3: we use branches A and B as two inputs and branch C as the output (Figure 4). To design a NOT gate (Figure 4(a)), we place 6 MRPs (a high iron volume fraction 18.48% and a small pillar spacing *L*/*D* = 1.5) at the central location of the channel. Effectively, 2 rows of MRP arrays are located on the pathway of A-to-C and 2 rows of MRP arrays on the pathway of B-to-C (Figure 4(b)). According to the results shown in Figure 3(d), (h), and (l), the acoustic transmission through one row of MRP array is ~0.35 at 8760 Hz. When input A and input B are both strong signals (normalized acoustic pressure amplitude 1), the output C behind two rows of MRP arrays is expected to feature a normalized pressure of (0.35 + 0.35) × 0.35 ≈ 0.25 at 8760 Hz (less than 0.5) (Figure 4(c)). We here denote that the normalized pressure equal to or larger than 0.5 as the digital “1” (strong) and otherwise as the digital “0” (weak). Numerical simulations show that the acoustic pressure amplitude at the output channel C is much smaller than the input channels A and B at 8760 Hz (Figure 4(d) and [Supplementary-material supplementary-material-1]). Experiments show that the normalized pressures in output C are all less than 0.5 whenever the inputs A and/or B are strong with a normalized pressure “1” or weak with a normalized pressure “0.2” at 8760 Hz (Figures 4(e) and [Supplementary-material supplementary-material-1]).

To switch the NOT gate to an AND gate (Figure 4(f)), one row of MRP array is bent by a magnetic field (i.e., 0.13 T), and thus, only one row of MRP array exists on the pathway of both A-to-C and B-to-C (Figure 4(g)). Since the acoustic transmission (normalized pressure) through one row of MRP array is ~0.35 at 8760 Hz, the normalized pressure in output C is expected to couple the signals from inputs A and B, leading to a normalized pressure of 0.35 + 0.35 = 0.7 (Figure 4(h)). This point can be validated by numerical simulations which show that the normalized pressure amplitude at output C is around 0.6-0.75 at 8760 Hz (Figure 4(i) and [Supplementary-material supplementary-material-1]). Experiments show that the normalized pressure at output C is ~0.6 (above 0.5 and marked as “1”) when the inputs A and B are both strong (normalized pressure “1”) at 8760 Hz (Figures 4(j) and [Supplementary-material supplementary-material-1]). To further switch the AND gate to an OR gate (Figure 4(k)), we use three magnets to bend 6 MRPs in the center of the channel (Figure 4(l) and [Supplementary-material supplementary-material-1]). Numerical simulations show that acoustic wave propagation to the output channel C does not have evident resistance at 8760 Hz (Figure 4(n) and [Supplementary-material supplementary-material-1]). Experiments show that the normalized pressure at output C is above 0.5 when either the input A or the input B is strong (normalized pressure “1”) at 8760 Hz (Figure 4(o) and [Supplementary-material supplementary-material-1]). Note that the above three acoustic logic operators can be on-demand and reversibly switched by controlling the untethered magnetic fields. Additional analyses and numerical studies show that the magnetoactive reconfigurable logic gates should work not only at 8760 Hz but also through 8700-8830 Hz and 8930-9070 Hz (Figs. [Supplementary-material supplementary-material-1], more discussion in Supplementary Information). This point is further confirmed by experiments at 8700 and 9050 Hz which are located at two frequency branches, respectively (Fig. [Supplementary-material supplementary-material-1]).

## 7. Magnetoactive Reconfigurable Acoustic Diode

Finally, we harness the MRPs to design a magnetoactive reconfigurable acoustic diode to smartly switch the reciprocity. Existing acoustic diodes or rectifiers that exhibit nonreciprocal acoustic wave propagation typically rely on acoustic metastructures with time-varying elements or nonlinearity [21–25, 50, 51]. An acoustic diode that can be switched by untethered stimuli has not been reported. Here, we harness untethered magnetic fields to demonstrate on-demand switching between the nonreciprocal acoustic diode and the reciprocal acoustic conductor.

**Figure 5:**
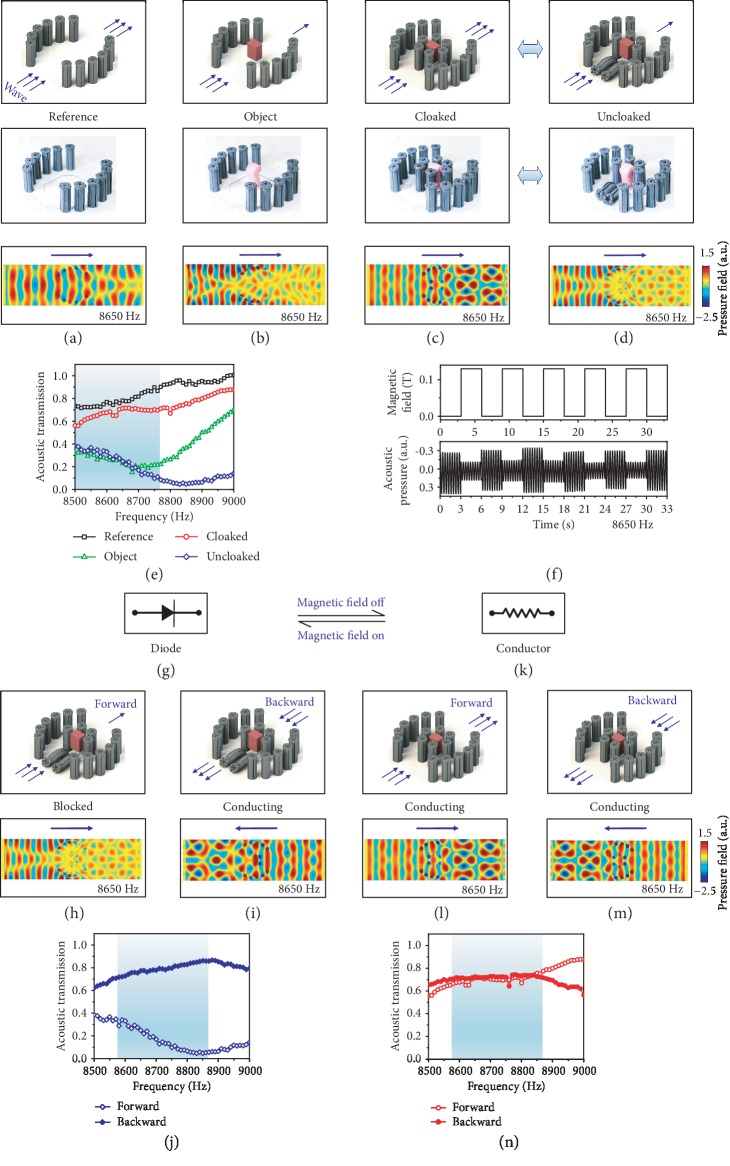
Magnetoactive reconfigurable acoustic diode. (a–d) Schematics, samples, and numerical simulations at 8650 Hz of four states: reference state (a), object state (b), cloak-like state (c), and blocked state (d). The object used is a cotton-covered plastic block. The selective actuation of two pillars in (d) is because of their higher iron volume fraction (18.48% by weight). (e) The acoustic transmission of four states in functions of the frequency. (f) The evolution of the transmitted acoustic pressure at the cloak-like and blocked states at 8650 Hz with cyclically switching magnetic fields. (g) Schematic of a diode. (h, i) Schematics and numerical simulations to show the acoustic wave propagation of the magnetically actuated (i.e., 0.13 T) acoustic diode in the forward (h) and backward (i) directions. (j) The experimentally measured acoustic transmissions of the acoustic diode in the forward and backward directions. (k) Schematic of a diode. (l, m) Schematics and numerical simulations to show the acoustic wave propagations of the acoustic conductor in the forward (l) and backward (m) directions. (n) The experimentally measured acoustic transmissions of the acoustic conductor in the forward and backward directions.

The idea for the acoustic diode is based on a cloak-like waveguide (Figure 5(a)–(d)). When an engineering object is placed on the pathway of an acoustic wave, the acoustic pressure amplitude behind the object is significantly reduced due to the refection or absorption of the object (Figure 5(a), (b), [Supplementary-material supplementary-material-1]). Experimental results show that the acoustic transmission within 8500-8800 Hz is reduced by more than a factor of 2 when the object is placed (Figure 5(b)). When the object is surrounded by 6 MRPs with spacing *L*/*D* = 1.5, the direct interaction between the incoming wave and the object is shielded by the MRPs within a certain Mie resonance frequency (e.g., 8500-8760 Hz), and the wave is guided by a circular pathway (spacing *L*/*D* ≥ 2.5) between the 6 central MRPs and the outside curved MRP arrays (Figure 5(c)). Numerical simulations show that the acoustic pressure amplitude behind the object increases drastically after the 6-MRP envelope is installed (8650 Hz, Figures 5(c) and [Supplementary-material supplementary-material-1]). Experiments show that the acoustic transmission of the wave is around 80-90% of that of the reference state without the object over frequency range of 8500-8760 Hz (Figure 5(e)). Note that the waveguide at the cloak-like state is not a perfect acoustic cloak because the incident acoustic mode is disrupted by the structure [52]. Interestingly, when the front two MRPs are bent by a magnetic field (i.e., 0.13 T), the cloak-like state is destroyed because the incoming acoustic wave can have direct interaction with the object, thus leading to a significantly low acoustic transmission within 8500-9000 Hz (Figure 5(d), (e), and [Supplementary-material supplementary-material-1]). The numerical simulations show that the acoustic amplitude behind the object drastically reduces after bending the front MRPs (Figure 5(d)), with the acoustic pressure field similar to that of the object state shown in Figure 5(b). Figure 5(e) shows the acoustic transmission of four interesting states within 8500-8760 Hz: the cloak-like and the reference states exhibit high acoustic transmission, and the blocked and object states exhibit low acoustic transmission. The experimentally measured acoustic transmissions of the four states within 8500-9000 Hz roughly agree with the respective numerical simulations (Fig. [Supplementary-material supplementary-material-1]). Besides, it should be noted that the cloak-like state (Figure 5(c)) and the blocked state (Figure 5(d)) can be reversibly and cyclically switched by controlling the magnetic field (Figure 5(f), [Supplementary-material supplementary-material-1]).

The acoustic diode (Figure 5(g)) is further designed based on the waveguide shown in Figure 5(a)–(d). The acoustic transmission of the MRP array with an applied magnetic field (i.e., 0.13 T) is relatively low (<0.4) within 8500-9000 Hz in the forward wave direction (Figure 5(h)). However, the acoustic transmission increases drastically to 0.7-0.8 within 8580-9000 Hz in the backward wave direction (Figure 5(i)), because the wave is guided by the MRPs to move around the object. The nonreciprocal wave propagation is visualized by the numerical simulations (Figure 5(h) and (i)) and validated by the experimentally measured acoustic transmissions in two directions (Figure 5(j)). The experimentally measured acoustic transmissions in two directions within 8500-9000 Hz agree with the numerical simulations (Fig. [Supplementary-material supplementary-material-1]). Effectively, the acoustic diode demonstrated here resembles the reported acoustic diodes that are composed of a sonic crystal with its own band structure and a nonlinear medium to destroy the system symmetry [22, 23, 25]. In this system, the MRPs define the band structure and the interactions between the incoming wave and the encapsulated object serve as the nonlinear element. Note that the acoustic mode has been changed during the transmission through the diode structure. It is possible due to the structural complexity, similar to the reported acoustic diode by Liang et al. [23, 25], where the highly nonlinear microbubble medium may also change the acoustic mode.

When the magnetic field is reduced or turned off, the bent MRPs become vertical and the structure turns back to a state of the relatively high acoustic transmission (~0.7) in both directions within 8580-8860 Hz because the waves are guided in both directions (Figure 5(k)–(n)). Therefore, on-demand tuning the applied magnetic field can enable reversible switching between a nonreciprocal acoustic diode (Figure 5(g)–(j)) and a reciprocal acoustic conductor (Figure 5(k)–(n)) within 8580-8860 Hz.

## 8. Discussion

In summary, we report a class of active acoustic metamaterials whose configurations can be on-demand switched via untethered magnetic fields, thus enabling active switching of acoustic transmission, wave guiding, logic operation, and reciprocity. The mechanism primarily relies on synergistic integration of MRP arrays and their large deformation actuated by magnetic fields. The MRP arrays allow a large freedom for constructing various acoustic metadevices (e.g., waveguide, logic gate, and diode) via the judicious design of the MRP layout. The magnetically induced large deformation of MRPs allows the configuration modulation and thus leads to function switching of the acoustic metadevices without fabricating new structures. This study highlights a unique paradigm for applying stimuli-responsive smart materials to acoustic metamaterials and metadevices to enable active control of their acoustic properties [32, 33]. This paradigm may promote the integration between various smart or soft materials and acoustic metamaterials to achieve unprecedented functionalities [53, 54]. The unique paradigm may also promote the study of active acoustic metamaterials in switching of acoustic properties via other untethered stimuli, such as electric field, light, and temperature. Besides, as the first generation of untethered-stimuli-induced active acoustic metadevices (including waveguide, logic gate, and diode), these active acoustic metadevices may be used in broad engineering settings, ranging from on-demand noise control in smart infrastructures, automobile, and aircraft [55] and audio modulation for the next generation sound devices [56] to sonic camouflage [13–15]. Furthermore, the concept of the active acoustic metamaterials enabled by magnetoactive structures may be extended to design various switchable acoustic metadevices. For example, the magnetoactive elastomers can be used to design twisted helicoids to design orbital angular momentum metastructures [57] which can be tuned on and off via magnetically modulating the axial lengths. As another example, magnetoactive acoustic tunneling may be realized by assembling magnetoactive Mie resonator arrays into the tunnel, thus enabling actively switching between the tunneling with supercoupling and regular wave guiding [58]. Likewise, the paradigm may be extended to other active acoustic metadevices, such as superlenses [6–12] and topological insulators [20, 26–29, 59, 60].

## 9. Materials and Methods

### 9.1. Sample Preparation

Mie resonator pillars (MRPs) were fabricated with a molding process usingadditive manufacturing technique (Fig. [Supplementary-material supplementary-material-1]). Molds were made with a fused deposition modeling (FDM) method with either ABS or dissolvable filament. The elastomer compound used for MRP is a mixture of 19.6 ml of liquid silicon rubber with various grams (5-35 g, Mold Max, Smooth-on) of iron nanoparticles (Sigma) ([Supplementary-material supplementary-material-1]). After mixing the liquid rubber evenly with the iron nanoparticles for 3-5 minutes, the mixture was slowly filled in the molds. Once the elastomer mixture was cured for 4 to 5 hours, the molds were either dissolved with solvent, d-limonene, or manually dissembled. When the solvent was used, the elastomer structure was dried on the hot plate (60°C) overnight. Then, a thin elastomer strap (1-2 mm) was added around the top of the elastomer pillar to prevent the spreading or opening of MRPs due to their own weight. In each experiment, we attach the MRPs on the testing platform with a superglue (Gorilla).

### 9.2. Magnetic Actuation

We put a magnet under the testing platform, yet 20-30 mm ahead of the MRP. This applies a diagonal magnetic field to the MRP. The magnetic field intensity was changed by moving the magnet upward. When the distance between the magnet and the MRPs becomes small enough, the MRPs pinched on to the substrate. Once the magnet was moved downward or removed, the bent MRPs returned to the upright position.

### 9.3. Measurement of Acoustic Transmission

Acoustic experiments were conducted in testing chambers with acoustic insulators (cotton pads) on the chamber walls. Two loudspeakers (OT19NC00-04, Tymphany) controlled by a function generator (PI-8127, PASCO, USA) were placed in the chambers to provide the acoustic signals. The acoustic signals were collected by microphones (378B02 with 426E01, PCB Piezotronics, USA) and processed by a signal conditioner (482C05, PCB Piezotronics) and displayed by an oscilloscope (TBS1052B, Tektronix, USA). The noise was further removed with Savitzky–Golay filter in MATLAB to calculate the acoustic amplitude. The acoustic transmission was measured as |*P*_*w*_/*P*_*wo*_|, where *P*_*w*_ and *P*_*wo*_ are acoustic amplitudes with and without the metastructures, respectively. The transmission measurement averaged over 2-4 measuring spots along a line normal to the wave propagation direction.

### 9.4. Acoustic Simulation

Numerical simulations were implemented with the acoustic module in COMSOL Multiphysics v5.2—a commercial finite element software. The set-ups for the numerical simulations are illustrated in Figs. [Supplementary-material supplementary-material-1], [Supplementary-material supplementary-material-1], [Supplementary-material supplementary-material-1], and [Supplementary-material supplementary-material-1]. In a typical simulation, three material phases were involved in the numerical models: elastomer with high iron volume fraction (18.48%), elastomer with low iron volume fraction (3.14%), and air. The densities and sound speeds of the elastomers are shown in [Supplementary-material supplementary-material-1]. Perfectly matching layers were employed to ensure the open boundary along the acoustic transport direction. The simulation was validated by benchmark calculations, and the mesh accuracy was ascertained through a mesh refinement study. The acoustic transmission was calculated by dividing the averaged acoustic pressure amplitude by the averaged background pressure amplitude over a line normal to the wave propagation direction.

## Supplementary Materials

Supplementary Materials Analogy between sharkskin denticles and Mie resonator pillars (MRPs). Modeling of MRPs. Theoretical analysis of magnetically induced buckling. Design principle of the reconfigurable acoustic logic gates. Table S1: experimentally measured materials and geometrical parameters of MRPs. Figure S1: fabrication process of MRPs. Figure S2: simulations of single MRP and MRP array. Figure S3: band structure of an MRP. Figure S4: theoretical analysis of an MRP. Figure S5: control solid pillar array. Figure S6: schematic for the placement of the magnet. Figure S7: schematic for a bottom-filed magnetoactive beam. Figure S8: simulation of the acoustic transmission of MRP arrays at two states. Figure S9: simulation configurations of the magnetoactive double-throw switch. Figure S10: effect of pillar spacing on acoustic transmission. Figure S11: simulation configurations of the magnetoactive logic gates. Figure S12: experimental results for logic gates. Figure S13: schematic for the magnet placement for logic gates. Figures S14-S15: simulations to reveal the mechanism for the acoustic logic gates. Figure S16: experimental results for logic gates at 8700 Hz and 9050 Hz. Figure S17: simulation configurations for the magnetoactive acoustic cloak-like waveguide. Figures S18-S19: simulations for the acoustic diode. Movie S1: magnetically induced cyclic bending of the central two pillars of an MRP array (period 5 s). Movie S2: magnetically induced cyclic bending of the central two pillars of an MRP array (period 1 s). Movie S3: magnetically induced cyclic bending of the front two pillars within an MRP-enabled cloak-like waveguide.

## References

[1] Ma G., Sheng P. (2016). Acoustic metamaterials: from local resonances to broad horizons. *Science Advances*.

[2] Cummer S. A., Christensen J., Alù A. (2016). Controlling sound with acoustic metamaterials. *Nature Reviews Materials*.

[3] Yang M., Sheng P. (2017). Sound absorption structures: from porous media to acoustic metamaterials. *Annual Review of Materials Research*.

[4] Hussein M. I., Leamy M. J., Ruzzene M. (2014). Dynamics of phononic materials and structures: historical origins, recent progress, and future outlook. *Applied Mechanics Reviews*.

[5] Assouar B., Liang B., Wu Y., Li Y., Cheng J.-C., Jing Y. (2018). Acoustic metasurfaces. *Nature Reviews Materials*.

[6] Kaina N., Lemoult F., Fink M., Lerosey G. (2015). Negative refractive index and acoustic superlens from multiple scattering in single negative metamaterials. *Nature*.

[7] Zhang S., Yin L., Fang N. (2009). Focusing ultrasound with an acoustic metamaterial network. *Physical Review Letters*.

[8] Spadoni A., Daraio C. (2010). Generation and control of sound bullets with a nonlinear acoustic lens. *Proceedings of the National Academy of Sciences of the United States of America*.

[9] Zhu J., Christensen J., Jung J. (2011). A holey-structured metamaterial for acoustic deep-subwavelength imaging. *Nature Physics*.

[10] Park C. M., Park J. J., Lee S. H., Seo Y. M., Kim C. K., Lee S. H. (2011). Amplification of acoustic evanescent waves using metamaterial slabs. *Physical Review Letters*.

[11] Li J., Fok L., Yin X., Bartal G., Zhang X. (2009). Experimental demonstration of an acoustic magnifying hyperlens. *Nature Materials*.

[12] Molerón M., Daraio C. (2015). Acoustic metamaterial for subwavelength edge detection. *Nature Communications*.

[13] Zhang S., Xia C., Fang N. (2011). Broadband acoustic cloak for ultrasound waves. *Physical Review Letters*.

[14] Zigoneanu L., Popa B.-I., Cummer S. A. (2014). Three-dimensional broadband omnidirectional acoustic ground cloak. *Nature Materials*.

[15] Popa B.-I., Zigoneanu L., Cummer S. A. (2011). Experimental acoustic ground cloak in air. *Physical Review Letters*.

[16] Zhang T., Cheng Y., Guo J. Z., Xu J. Y., Liu X. J. (2015). Acoustic logic gates and Boolean operation based on self-collimating acoustic beams. *Applied Physics Letters*.

[17] Zhang T., Cheng Y., Yuan B.-G., Guo J.-Z., Liu X.-J. (2016). Compact transformable acoustic logic gates for broadband complex Boolean operations based on density-near-zero metamaterials. *Applied Physics Letters*.

[18] Li F., Anzel P., Yang J., Kevrekidis P. G., Daraio C. (2014). Granular acoustic switches and logic elements. *Nature Communications*.

[19] Wang Y., Xia J. P., Sun H. X., Yuan S. Q., Liu X. J. (2019). Binary-phase acoustic passive logic gates. *Scientific Reports*.

[20] Xia J. P., Jia D., Sun H. X. (2018). Programmable coding acoustic topological insulator. *Advanced Materials*.

[21] Popa B.-I., Cummer S. A. (2014). Non-reciprocal and highly nonlinear active acoustic metamaterials. *Nature Communications*.

[22] Boechler N., Theocharis G., Daraio C. (2011). Bifurcation-based acoustic switching and rectification. *Nature Materials*.

[23] Liang B., Guo X. S., Tu J., Zhang D., Cheng J. C. (2010). An acoustic rectifier. *Nature Materials*.

[24] Fleury R., Sounas D. L., Sieck C. F., Haberman M. R., Alù A. (2014). Sound isolation and giant linear nonreciprocity in a compact acoustic circulator. *Science*.

[25] Liang B., Yuan B., Cheng J. C. (2009). Acoustic diode: rectification of acoustic energy flux in one-dimensional systems. *Physical Review letters*.

[26] Fleury R., Khanikaev A. B., Alu A. (2016). Floquet topological insulators for sound. *Nature Communications*.

[27] Peng Y.-G., Qin C. Z., Zhao D. G. (2016). Experimental demonstration of anomalous Floquet topological insulator for sound. *Nature Communications*.

[28] He C., Ni X., Ge H. (2016). Acoustic topological insulator and robust one-way sound transport. *Nature Physics*.

[29] Yang Z., Gao F., Shi X. (2015). Topological acoustics. *Physical Review Letters*.

[30] Babaee S., Viard N., Wang P., Fang N. X., Bertoldi K. (2016). Harnessing deformation to switch on and off the propagation of sound. *Advanced Materials*.

[31] Babaee S., Overvelde J. T. B., Chen E. R., Tournat V., Bertoldi K. (2016). Reconfigurable origami-inspired acoustic waveguides. *Science Advances*.

[32] Kadic M., Milton G. W., van Hecke M., Wegener M. (2019). 3D metamaterials. *Nature Reviews Physics*.

[33] Bertoldi K., Vitelli V., Christensen J., van Hecke M. (2017). Flexible mechanical metamaterials. *Nature Reviews Materials*.

[34] Wang P., Casadei F., Shan S., Weaver J. C., Bertoldi K. (2014). Harnessing buckling to design tunable locally resonant acoustic metamaterials. *Physical Review Letters*.

[35] Yu K., Fang N. X., Huang G., Wang Q. (2018). Magnetoactive acoustic metamaterials. *Advanced Materials*.

[36] Xiao S., Ma G., Li Y., Yang Z., Sheng P. (2015). Active control of membrane-type acoustic metamaterial by electric field. *Applied Physics Letters*.

[37] Cheng Y., Zhou C., Yuan B. G., Wu D. J., Wei Q., Liu X. J. (2015). Ultra-sparse metasurface for high reflection of low-frequency sound based on artificial Mie resonances. *Nature Materials*.

[38] Liang Z., Li J. (2012). Extreme acoustic metamaterial by coiling up space. *Physical Review Letters*.

[39] Xie Y., Popa B.-I., Zigoneanu L., Cummer S. A. (2013). Measurement of a broadband negative index with space-coiling acoustic metamaterials. *Physical Review Letters*.

[40] Ball P. (1999). Engineering shark skin and other solutions. *Nature*.

[41] Wen L., Weaver J. C., Lauder G. V. (2014). Biomimetic shark skin: design, fabrication and hydrodynamic function. *The Journal of Experimental Biology*.

[42] Dean B., Bhushan B. (2010). Shark-skin surfaces for fluid-drag reduction in turbulent flow: a review. *Philosophical Transactions of the Royal Society A: Mathematical, Physical and Engineering Sciences*.

[43] Bechert D. W., Bruse M., Hage W., van der Hoeven J. G. T., Hoppe G. (1997). Experiments on drag-reducing surfaces and their optimization with an adjustable geometry. *Journal of Fluid Mechanics*.

[44] Domel A. G., Saadat M., Weaver J. C., Haj-Hariri H., Bertoldi K., Lauder G. V. (2018). Shark skin-inspired designs that improve aerodynamic performance. *Journal of the Royal Society Interface*.

[45] Oeffner J., Lauder G. V. (2012). The hydrodynamic function of shark skin and two biomimetic applications. *Journal of Experimental Biology*.

[46] Brunet T., Merlin A., Mascaro B. (2015). Soft 3D acoustic metamaterial with negative index. *Nature Materials*.

[47] Zhu X., Liang B., Kan W., Peng Y., Cheng J. (2016). Deep-subwavelength-scale directional sensing based on highly localized dipolar Mie resonances. *Physical Review Applied*.

[48] Ying C., Xiao-Jun L. (2009). Extraordinary resonant scattering in imperfect acoustic cloak. *Chinese Physics Letters*.

[49] Hu X., Ho K.-M., Chan C. T., Zi J. (2008). Homogenization of acoustic metamaterials of Helmholtz resonators in fluid. *Physical Review B*.

[50] Shen Y.-X., Peng Y.-G., Zhao D.-G., Chen X.-C., Zhu J., Zhu X.-F. (2019). One-way localized adiabatic passage in an acoustic system. *Physical Review Letters*.

[51] Zhu X., Zou X., Liang B., Cheng J. (2010). One-way mode transmission in one-dimensional phononic crystal plates. *Journal of Applied Physics*.

[52] Esfahlani H., Karkar S., Lissek H., Mosig J. R. (2016). Acoustic carpet cloak based on an ultrathin metasurface. *Physical Review B*.

[53] Shen Y.-X., Peng Y. G., Cai F. (2019). Ultrasonic super-oscillation wave-packets with an acoustic meta-lens. *Nature Communications*.

[54] Tang H., Chen Z., Tang N. (2018). Hollow-out patterning ultrathin acoustic metasurfaces for multifunctionalities using soft fiber/rigid bead networks. *Advanced Functional Materials*.

[55] Martínez-Sala R., Sancho J., Sánchez J. V., Gómez V., Llinares J., Meseguer F. (1995). Sound attenuation by sculpture. *Nature*.

[56] Xie Y., Tsai T.-H., Konneker A., Popa B.-I., Brady D. J., Cummer S. A. (2015). Single-sensor multispeaker listening with acoustic metamaterials. *Proceedings of the National Academy of Sciences of the United States of America*.

[57] Esfahlani H., Lissek H., Mosig J. R. (2017). Generation of acoustic helical wavefronts using metasurfaces. *Physical Review B*.

[58] Esfahlani H., Byrne M. S., McDermott M., Alù A. (2019). Acoustic supercoupling in a zero-compressibility waveguide. *Research*.

[59] Ding Y., Peng Y., Zhu Y. (2019). Experimental demonstration of acoustic Chern insulators. *Physical Review Letters*.

[60] Peng Y.-G., Li Y., Shen Y. X. (2019). Chirality-assisted three-dimensional acoustic Floquet lattices. *Physical Review Research*.

