# Supplementary material for: Sharkskin-Inspired Magnetoactive Reconfigurable Acoustic Metamaterials
Source: Research (Wash D C). 2020 Feb 5;2020:4825185. doi: 10.34133/2020/4825185 (PMC7025040; doi:10.34133/2020/4825185)
Supplement: Supplementary Materials — Analogy between sharkskin denticles and Mie resonator pillars (MRPs). Modeling of MRPs. Theoretical analysis of magnetically induced buckling. Design principle of the reconfigurable acoustic logic gates. Table S1: experimentally measured materials and geometrical parameters of MRPs. Figure S1: fabrication process of MRPs. Figure S2: simulations of single MRP and MRP array. Figure S3: band structure of an MRP. Figure S4: theoretical analysis of an MRP. Figure S5: control solid pillar array. Figure S6: schematic for the placement of the magnet. Figure S7: schematic for a bottom-filed magnetoactive beam. Figure S8: simulation of the acoustic transmission of MRP arrays at two states. Figure S9: simulation configurations of the magnetoactive double-throw switch. Figure S10: effect of pillar spacing on acoustic transmission. Figure S11: simulation configurations of the magnetoactive logic gates. Figure S12: experimental results for logic gates. Figure S13: schematic for the magnet placement for logic gates. Figures S14-S15: simulations to reveal the mechanism for the acoustic logic gates. Figure S16: experimental results for logic gates at 8700 Hz and 9050 Hz. Figure S17: simulation configurations for the magnetoactive acoustic cloak-like waveguide. Figures S18-S19: simulations for the acoustic diode. Movie S1: magnetically induced cyclic bending of the central two pillars of an MRP array (period 5 s). Movie S2: magnetically induced cyclic bending of the central two pillars of an MRP array (period 1 s). Movie S3: magnetically induced cyclic bending of the front two pillars within an MRP-enabled cloak-like waveguide. [file 4825185.f1.pdf]

Supplementary Information for

Sharkskin-Inspired Magnetoactive Acoustic Metasurfaces

Kyung Hoon Lee, Kunhao Yu, Hasan Al Ba'ba'a, An Xin, Zhangzhengrong Feng, Qiming Wang*

Sonny Astani Department of Civil and Environmental Engineering, University of Southern California, Los Angeles, CA 90089, USA

*E-mail: qimingw@usc.edu

1. Analogy between sharkskin denticles and Mie resonator pillars

We use the metaphor of sharkskin because of the analogy in following 3 aspects:

1) Pillar-like structures: The sharkskin features pillar-like denticle arrays on its surface[1-3]. In this paper we employ Mie resonator pillar arrays.
2) Function in actively switching flow transmission: The sharkskin can smartly switch the surface fluid flow transmission[4]. This paper is to actively switch the acoustic flow transmission.
3) Actuation via tilting pillars: The modulation of flow drag of the sharkskin is through tilting the pillar-like denticles[2, 5, 6]. The modulation of acoustic transmission in this paper is through magnetically-induced pillar bending/tilting.

2. Modeling of the Mie resonator pillar (MRP)

Analytical modeling. Here, we estimate the resonance of the MRP based on a homogenization procedure presented in Reference[7]. For the MRP in **Fig. S4a**, the medium can be simplified into six channels (**Fig. S4b**), which are shorter than a wavelength[8]. Such a model can then be homogenized and equivalently represented as three two-dimensional regions with different effective properties as seen in **Fig. S4c**. The pressure fields in the different regions, denoted here as *I*, *II* and *III*, are expressed mathematically by a combination of Bessel function J_m and Hankel function H_m as[7, 9]:

$$P^{I}(r) = \sum_m G_m J_m(k_0 r) e^{im\theta}, r < r_2 \tag{S1}$$

$$P^{II}(r) = \sum_m [E_m J_m(k_1 r) + F_m H_m(k_1 r)] e^{im\theta}, r_2 < r < r_1 \tag{S2}$$

$$P^{III}(r) = \sum_m [c_m J_m(k_0 r) + D_m H_m(k_0 r)] e^{im\theta}, r > R \tag{S3}$$

where $R = D/2$ is the total radius of the individual resonator, r_2 is the inner radius of the resonator housing the air core, and $r_1 = r_2 + (R - r_2)\eta$ is the outer radius of a uniform equivalent layer with η being the filling ratio of the air channels. In our case, the filling ratio η is approximately equal to 0.36. Note here that all the radii are measured from the center of the resonator, i.e. $r = 0$. To satisfy the continuity at the interfaces $r = r_2$ and $r = r_1$, the pressure field and $\frac{1}{\rho}\partial_r P(r)$ are matched at the boundaries[10], which gives two sets of conditions:

$$P^{I}(r_2) = P^{II}(r_2) \tag{S4}$$

$$\frac{1}{\rho_0}\partial_r P^{I}(r_2) = \frac{1}{\rho_1}\partial_r P^{II}(r_2) \tag{S5}$$

$$P^{II}(r_2) = P^{III}(R) \tag{S6}$$

$$\frac{1}{\rho_1}\partial_r P^{II}(r_2) = \frac{1}{\rho_0}\partial_r P^{III}(R) \tag{S7}$$

where $\rho_{0,1}$ denote the density of regions I, III and II, respectively. Consequently, one can get the following equations, for the mth term of the summation:

$$G_m J_m(k_0 r_2) - E_m J_m(k_1 r_2) - F_m H_m(k_1 r_2) = 0 \tag{S8}$$

$$\frac{k_0}{\rho_0} G_m \partial_r J_m(k_0 r_2) - \frac{k_1}{\rho_1}[E_m \partial_r J_m(k_1 r_2) + F_m \partial_r H_m(k_1 r_2)] = 0 \tag{S9}$$

$$c_m J_m(k_0 R) + D_m H_m(k_0 R) - E_m J_m(k_1 r_1) - F_m H_m(k_1 r_1) = 0 \tag{S10}$$

$$\frac{k_0}{\rho_0} c_m \partial_r J_m(k_0 R) + \frac{k_0}{\rho_0} D_m \partial_r H_m(k_0 R) - \frac{k_1}{\rho_1}[E_m \partial_r J_m(k_1 r_1) + F_m \partial_r H_m(k_1 r_1)] = 0 \tag{S11}$$

which can be cast into a compact matrix form:

$$\begin{bmatrix} J_m(k_0 r_2) & -J_m(k_1 r_2) & -H_m(k_1 r_2) & 0 \\ \frac{k_0}{\rho_0}\partial_r J_m(k_0 r_2) & -\frac{k_1}{\rho_1}\partial_r J_m(k_1 r_2) & -\frac{k_1}{\rho_1}\partial_r H_m(k_1 r_2) & 0 \\ 0 & -J_m(k_1 r_1) & -H_m(k_1 r_1) & H_m(k_0 R) \\ 0 & -\frac{k_1}{\rho_1}\partial_r J_m(k_1 r_1) & -\frac{k_1}{\rho_1}\partial_r H_m(k_1 r_1) & \frac{k_0}{\rho_0}\partial_r H_m(k_0 R) \end{bmatrix} \begin{Bmatrix} G_m \\ E_m \\ F_m \\ D_m \end{Bmatrix} = \begin{Bmatrix} 0 \\ 0 \\ -J_m(k_0 R) \\ -\frac{k_0}{\rho_0}\partial_r J_m(k_0 R) \end{Bmatrix} \tag{S12}$$

where the coefficients G_m through D_m are now normalized by the coefficient c_m. We assume that the effective uniform medium density has the same bulk modulus as the background medium, but different density defined as $\rho_1 = \rho_0/\eta^2$ [10]. Of interest here is the determination of scattering coefficient D_m. Using the scheme presented in reference[9] for calculating D_m via a physically revealing model, the scattering coefficient is found by:

$$D_m = -\frac{U_m}{U_m + i\,V_m} \tag{S13}$$

where i is the imaginary unit and U_m and V_m are defined as the following determinants:

$$U_m = \begin{vmatrix} J_m(k_0 r_2) & J_m(k_1 r_2) & Y_m(k_1 r_2) & 0 \\ \frac{k_0}{\rho_0}\partial_r J_m(k_0 r_2) & \frac{k_1}{\rho_1}\partial_r J_m(k_1 r_2) & \frac{k_1}{\rho_1}\partial_r Y_m(k_1 r_2) & 0 \\ 0 & J_m(k_1 r_1) & Y_m(k_1 r_1) & J_m(k_0 R) \\ 0 & \frac{k_1}{\rho_1}\partial_r J_m(k_1 r_1) & \frac{k_1}{\rho_1}\partial_r Y_m(k_1 r_1) & \frac{k_0}{\rho_0}\partial_r J_m(k_0 R) \end{vmatrix} \tag{S14}$$

$$V_m = \begin{vmatrix} J_m(k_0 r_2) & J_m(k_1 r_2) & Y_m(k_1 r_2) & 0 \\ \frac{k_0}{\rho_0}\partial_r J_m(k_0 r_2) & \frac{k_1}{\rho_1}\partial_r J_m(k_1 r_2) & \frac{k_1}{\rho_1}\partial_r Y_m(k_1 r_2) & 0 \\ 0 & J_m(k_1 r_1) & Y_m(k_1 r_1) & Y_m(k_0 R) \\ 0 & \frac{k_1}{\rho_1}\partial_r J_m(k_1 r_1) & \frac{k_1}{\rho_1}\partial_r Y_m(k_1 r_1) & \frac{k_0}{\rho_0}\partial_r Y_m(k_0 R) \end{vmatrix} \tag{S15}$$

where Y_m is the Bessel function of the second kind. Following the assumptions $k_0 R \ll 1$ and $k_0 r_2 \ll 1$, the first monopole frequency is found by setting $m = 0$ [7]. If the diameter of the MRP is 1.5 cm, the Mie resonance is estimated as $f_R \sim 8.9$ kHz, which is found from the frequency response of $|D_0|$ where it approaches 1, signaling the occurrence of a scattering resonance (**Fig. S4d**) [7]. The magnitude $|D_0|$ and angle of D_0 are depicted in **Figs. S4d, e**. This result is close to the experimentally observed and numerically simulated 9.1 kHz (**Fig. 2**).

Effect of geometrical inconsistency. If the fabrication of the MRP cannot accurately follow the geometrical design, the diameter of the MRP may have geometrical inconsistency. If the diameter of the MRP varies from 1.4 cm to 1.6 cm (7% of variation around 1.5 cm), the Mie resonance frequency changes from 9.48 kHz to 8.3 kHz (**Figs. S4d, e**). This variation of the Mie resonance frequency can fully cover the interesting frequency range in the experiments: 8600-9200 Hz. This theoretically predicted effect of the geometrical inconsistency also agrees well with the numerical simulations shown in **Fig. S2c**.

3. Theoretical analysis of magnetically-induced buckling

Following the previously reported work on magnetically-induced buckling of tilted beam[11], we here develop an analytical model for the magnetically-induced bending of the pillar. The magnetically-induced bending can be modeled as a tilted pillar under a magnetic field with an angle α to the pillar (**Fig. S7**). We construct two coordinate systems: global Cartesian coordinate (x,y) and local curvilinear coordinate (s,θ) shown in **Fig. S7**. The free energy of the magnetic-field deformed pillar can be written as[11-16]

$$\Pi = \int_0^H \frac{EI}{2}\left(\frac{d\theta}{ds}\right)^2 ds - \int_0^H A\left[\int_0^{\boldsymbol{B}} \boldsymbol{M}\cdot d\boldsymbol{B}\right] ds \qquad \text{(S16)}$$

where s is the curvilinear coordinate along the beam, θ is the angle between the tangent line and the horizontal axis, E is Young's modulus of the magnetoactive elastomer, H is the pillar length, I is the second moment of the cross-section area, A is the cross-section area, **B** is the applied magnetic field vector, and **M** is the magnetization vector.

We first assume that the pillar aspect ratio (H/D) is relatively large, and the pillar can be considered as a slender structure. The variation of **Eq. S16** leads to a governing equation of the magnetically-induced bending written as[11-16]

$$\lambda^2\left(\frac{d^2\theta}{ds^2}\right) + sin(2\theta + 2\alpha) = 0 \qquad \text{(S17)}$$

where $\lambda = \sqrt{2E\mu_0 I/(AB^2\Delta\chi)}$ is a characteristic length, $\mu_0 = 4\pi \times 10^{-7} N \cdot A^{-2}$ is the magnetic permittivity of the vacuum, and α is the initial tilted angle of the beam. $\Delta\chi$ is the effective magnetic susceptibility difference between the axial and orthogonal direction and can be estimated as $\Delta\chi \approx \chi - \chi/(1 + \chi/2)$, where χ is the magnetic susceptibility of the elastomer.

At the critical point of the buckling, the characteristic length λ should scale with the beam length H. Therefore, the critical magnetic field of the buckling should follow a scaling law as

$$B_c \propto \sqrt{\frac{\mu_0 E}{\Delta\chi}\left(\frac{I}{AH^2}\right)} \quad \text{(S18)}$$

Once the applied magnetic field is larger than the critical magnetic field for the pillar buckling, the pillar will be bent and pinched on the substrate.

According to **Eq. S18**, the critical magnetic field is affected by the Young's modulus, magnetic susceptibility, cross-section geometry, and the length of the pillar. Besides, we notice that the critical magnetic field should also be affected by the tilting angle α of the magnetic field. The effect of the tilting angle should be reflected by the pre-factor of the scaling law, β. In addition, **Eq. S18** is derived based on a slender assumption. If the slender condition is relaxed to extended to relatively small pillar aspect ratio (e.g., *H/D*=2.83 in this work), the contribution of the geometrical factor *H/D* should be also reflected by the pre-factor β. Therefore, the critical magnetic field of the buckling can be written as

$$B_c = \beta\left(\alpha, \frac{H}{D}\right)\sqrt{\frac{\mu_0 E}{\Delta\chi}\left(\frac{I}{AH^2}\right)} \quad \text{(S19)}$$

where the pre-factor is a function of tilting angle α and pillar aspect ratio H/D.

To validate the theoretical model in **Eq. S19**, we employ Mie resonator pillars (MRPs) with varied concentration of the ferromagnetic iron particle within the magnetoactive elastomer but maintain the geometry of the pillar and tilting angle $\alpha = \pi/4$. The variation of the iron concentration changes the Young's modulus and magnetic susceptibility of the magnetoactive elastomer. We measure the material and geometrical parameters of the MRPs and show them in **Table S2**. Experiments verify that the critical magnetic field indeed follows a linear relationship with $\sqrt{\frac{\mu_0 E}{\Delta\chi}\left(\frac{I}{AH^2}\right)}$ (**Fig. 2n**).

4. Design principle of the reconfigurable acoustic logic gates

The design of the switchable logic gate is based on two mechanisms:

(1) The transmission ratios of the MRP arrays decreases with the row number of the array. For example, the acoustic transmission through one row of MRP array is around 0.35 at 8760 Hz; thus, the acoustic transmission through two rows of MRP array is around 0.35^2=0.12 at 8760 Hz (**Figs. 4c, h, m,** and **S14**). If two inputs are applied, the output transmission of one-row MRP array is 0.35+0.35=0.7 (above 0.5), but the output transmission of two-row MRP array is only 0.12+0.12=0.24 (below 0.5). We here denote that the normalized pressure equal to or larger than 0.5 as "1" and otherwise as "0". Then, these two types of MRP array show different output states: the former outputs "1" but the latter outputs "0".

(2) The magnetic field can be used to bend a row of MRP array to leave only one-row MRP array. On-off switching the magnetic field can one-demand switch between one-row array and two-row array.

According to the design principle, to enable switching among NOT, AND, and OR operators, the acoustic transmission of one-row MRP array can be around 0.25-0.5. The corresponding frequency range is slightly below or above the Mie resonance frequency range, for example, 8700-8830 Hz and 8930-9070 Hz (according to data shown in **Fig. S14a**). To verify this working frequency range for the switching of three logic operators, we carry out numerical simulations that show the frequencies through 8700-8830 Hz and 8930-9070 Hz work well for the switching of three logic operators (**Figs. S14 and S15**). This point is also confirmed by experiments at 8700 and 9050 Hz which are located at two frequency branches, respectively (**Fig. S16**).

5. Supplementary tables

Table S1. Experimentally-measured materials and geometrical parameters of Mie resonator pillars. The sound speed within the filled elastomer is estimated by assuming the Poisson's ratio as 0.48.

Iron weight per 22 g elastomer	Iron volume fraction (%)	Young's modulus of filled elastomer (MPa)	Density of filled elastomer (kg/m^3)	Sound speed within filled elastomer (m/s)	Effective magnetic susceptibility difference $\Delta\chi$	Height H (cm)	Cross-section area of solid part (cm^2)	The second moment of area (cm^4)
5	3.14	0.22661	1019	43.05	0.169	4.25	1.102	0.1772
10	6.08	0.26147	1205	42.52	0.457			
15	8.85	0.28785	1307	42.84	0.953			
20	11.47	0.30889	1413	42.68	2.03			
25	13.94	0.36696	1675	42.73	6.02			
30	16.27	0.43402	1770	45.20	8.34			
35	18.48	0.49576	1860	47.13	11.53			

6. Supplementary figures

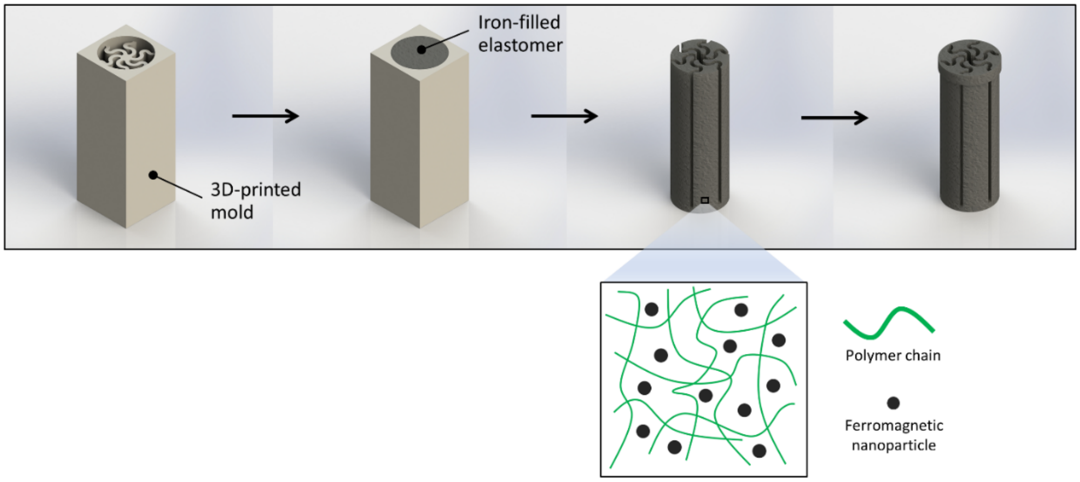

Figure S1. The fabrication process of a Mie resonator pillar.

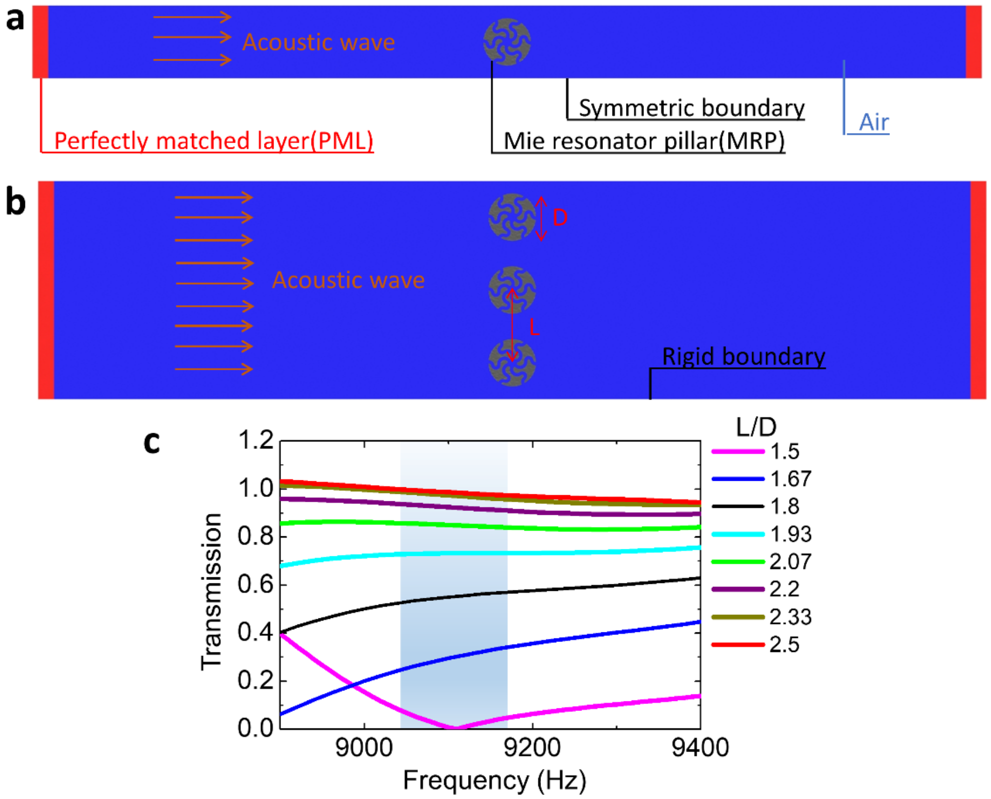

Figure S2. **(a)** Configuration of the numerical simulation for single Mie resonator pillar (MRP). **(b)** Configuration of the numerical simulation for an MRP array. **(c)** The acoustic transmission of the MRP arrays with various relative spacing L/D in functions of the frequency. Within frequency 9050-9150 Hz, the acoustic transmission increases with increasing pillar spacing.

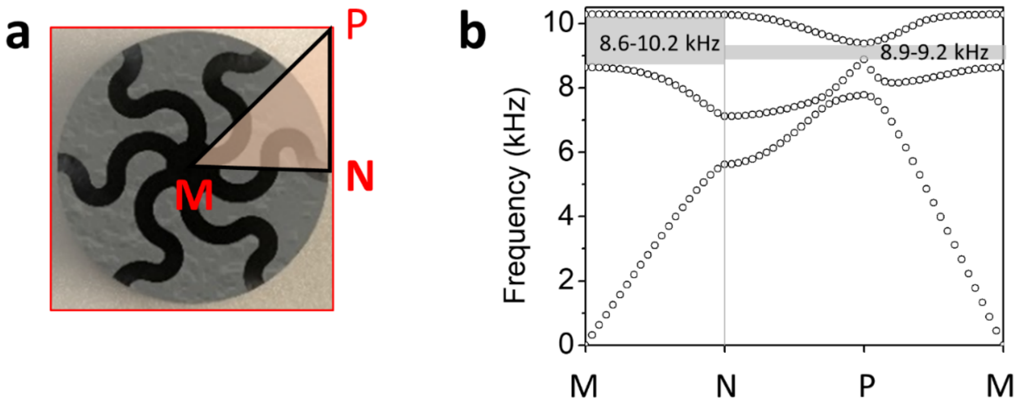

Figure S3. **(a)** Schematic to show the Brillion zone. **(b)** The band structure of the MRP. The MRP exhibits a bandgap within 8.6-10.2 kHz for the horizontal wave and a bandgap within 8.9-9.2 kHz for waves with all directions.

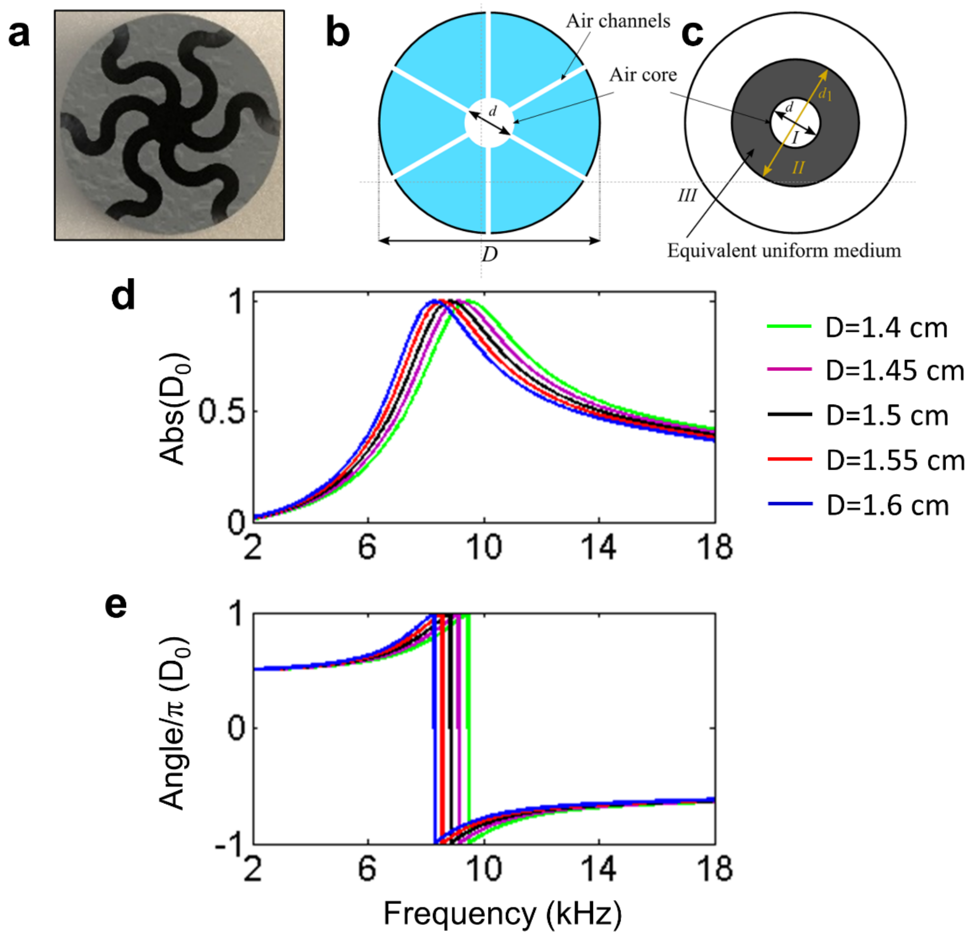

Figure S4. (a) A picture of the cross-sectional area of MRP. **(b)** The equivalent physical model with air channels with low-sound speed as well as its realization based on the homogenization procedure. **(c)** The equivalent model used for the analytical model. The equivalent media (gray area) is used for the analytical estimation of the Mie resonance with $d_1 = 2r_1$ being the outer diameter of the equivalent uniform medium. Note here that the white areas in b represent the background fluid medium (air). **(d, e)** Frequency sweep of the absolute value of the complex scattering coefficient, i.e. $|D_0|$, and its corresponding angle. The peak occurs at the scattering resonance, which approximately occurs at $f_R \approx 8.9$ kHz when D=1.5 cm. The resonance frequency varies from 9.48 kHz to 8.3 kHz when the diameter of the pillar D varies from 1.4 cm to 1.6 cm.

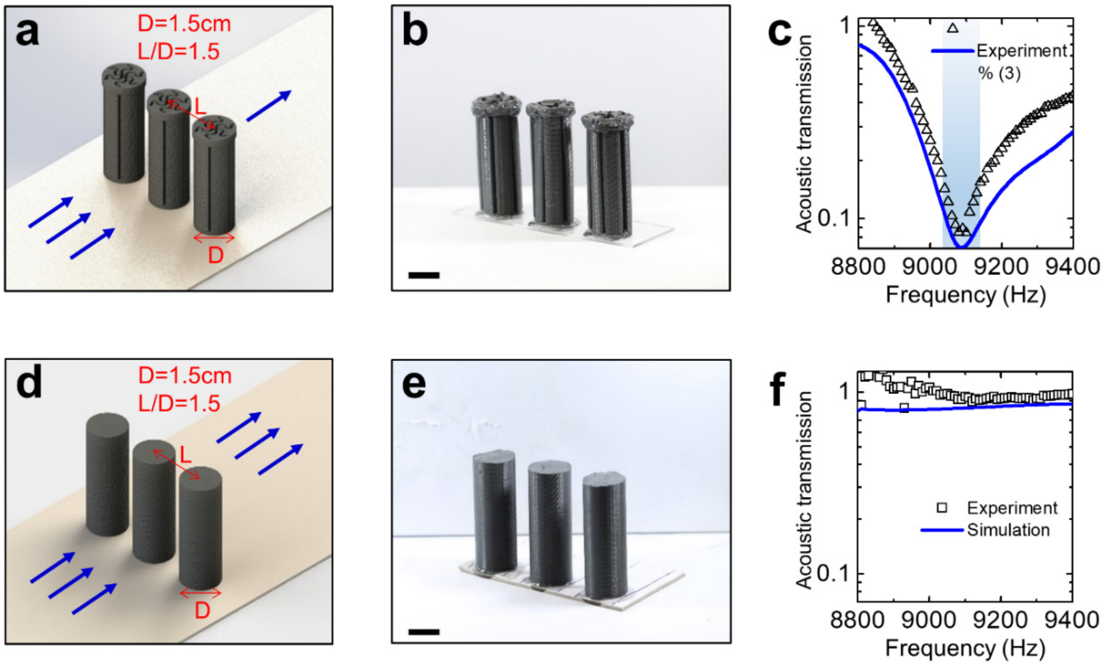

Figure S5. Schematics **(a, d)**, samples **(b, e)**, and transmission-frequency results **(c, f)** of MRP array **(a-c)** and solid pillar array **(d-f)** with the same pillar diameter and spacing. Scale bars in **b** and **e** denote 1 cm.

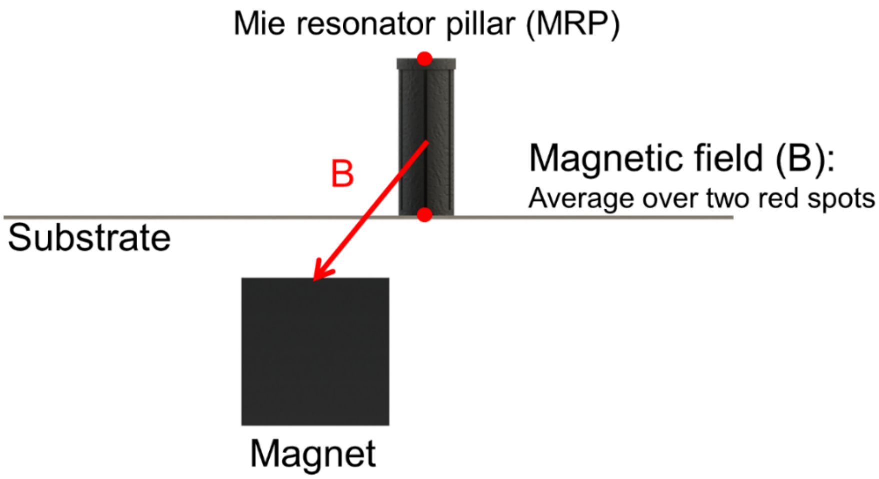

Figure S6. Schematic to show the application of the magnetic field to the MRP. The effective magnetic field is calculated by averaging the magnetic field at the bottom and the top of the MRP.

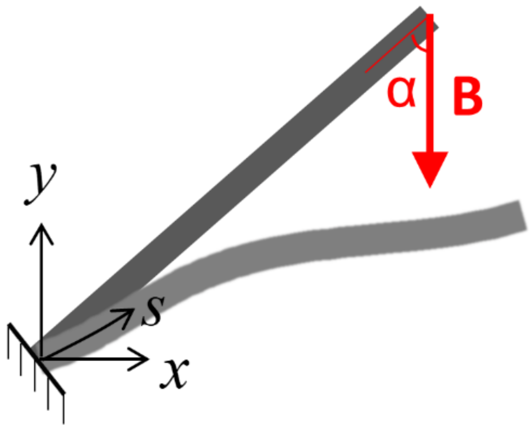

Figure S7. Schematic to show the deformation of a bottom-fixed magnetoactive beam under a tilted magnetic field.

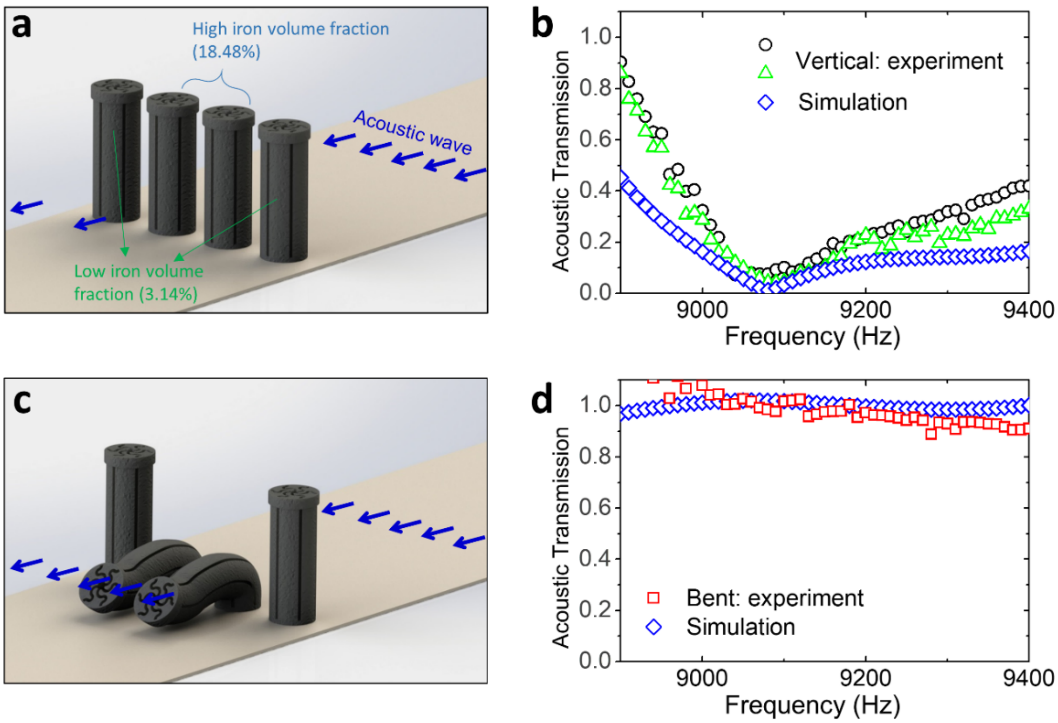

Figure S8. **(a)** Schematic to show the acoustic transportation through an MRP array with two pillars with high iron volume fraction 18.48% and two pillars with low iron volume fraction 3.14%. **(b)** The experimentally measured and numerically simulated acoustic transmissions of a in functions of the frequency. **(c)** Schematic to show the acoustic transportation through an MRP array with the central two pillars bent by a magnetic field. **(d)** The experimentally measured and numerically simulated acoustic transmissions of **c** in functions of the frequency.

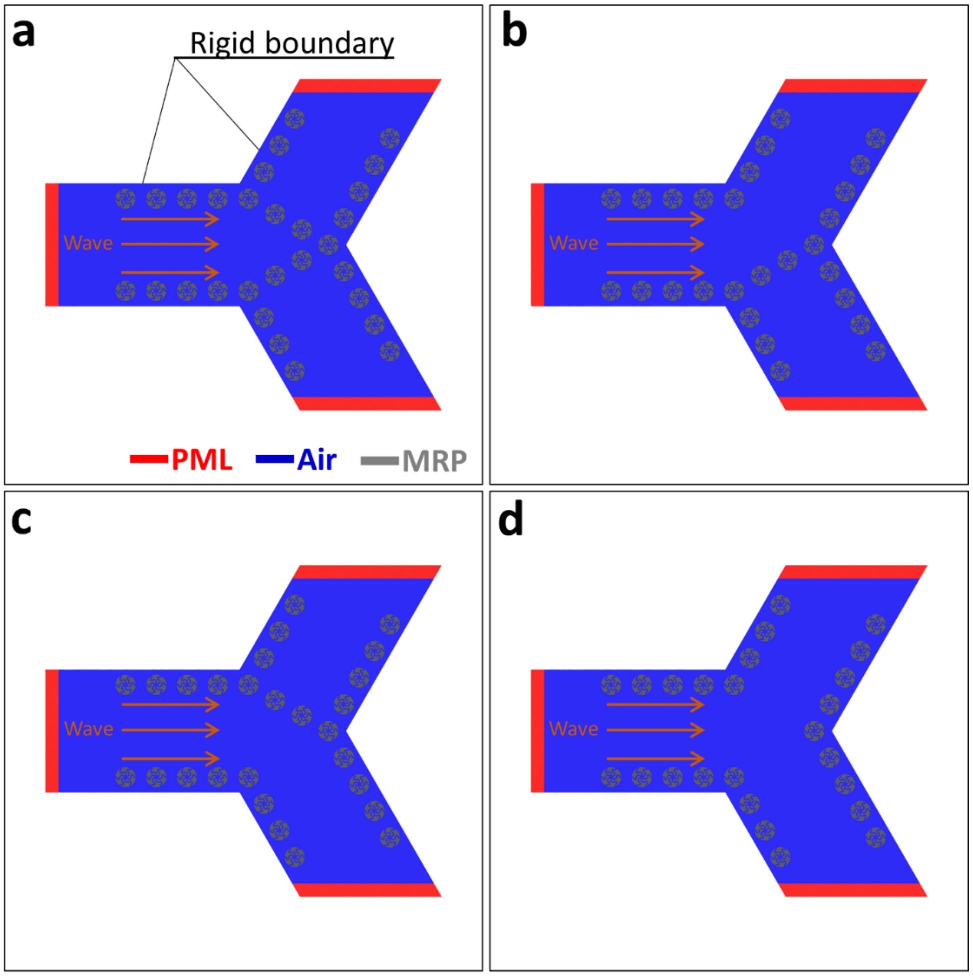

Figure S9. Configurations of the numerical simulations for the magnetoactive double-through switch shown in **Fig. 3**: **(a)** A off B off, **(b)** A on B off, **(c)** A off B on, and **(d)** A on B on. Note that the bent pillars in b-d are removed in the simulations.

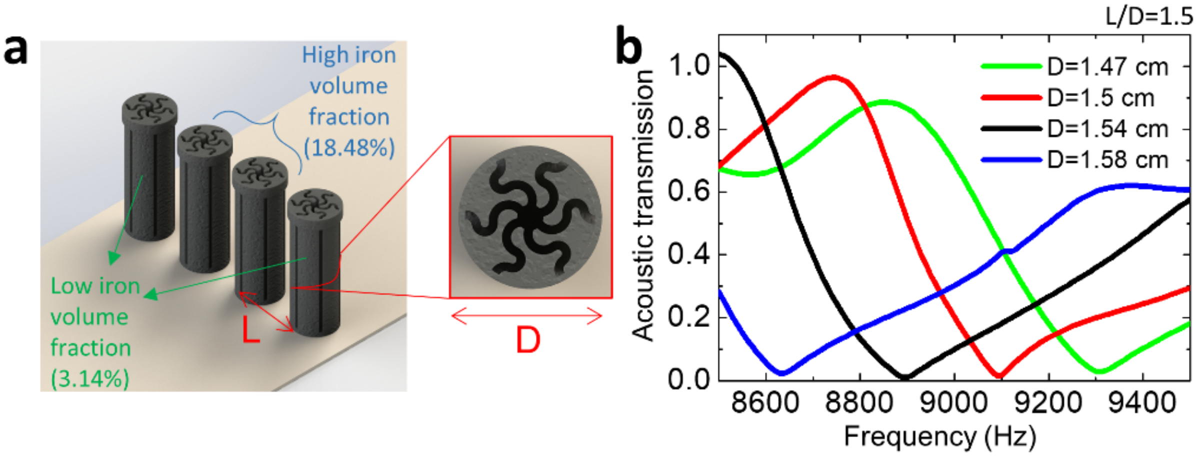

Figure S10. **(a)** Schematic for an MRP array with two pillars with high iron volume fraction 18.48% and two pillars with low iron volume fraction 3.14%. **(b)** The numerically simulated acoustic transmission through the MRP array shown in **(a)** with various relative spacings L/D. The Mie resonance frequency changes from 8620 to 9300 Hz when the relative spacing L/D changes from 1.47 to 1.58.

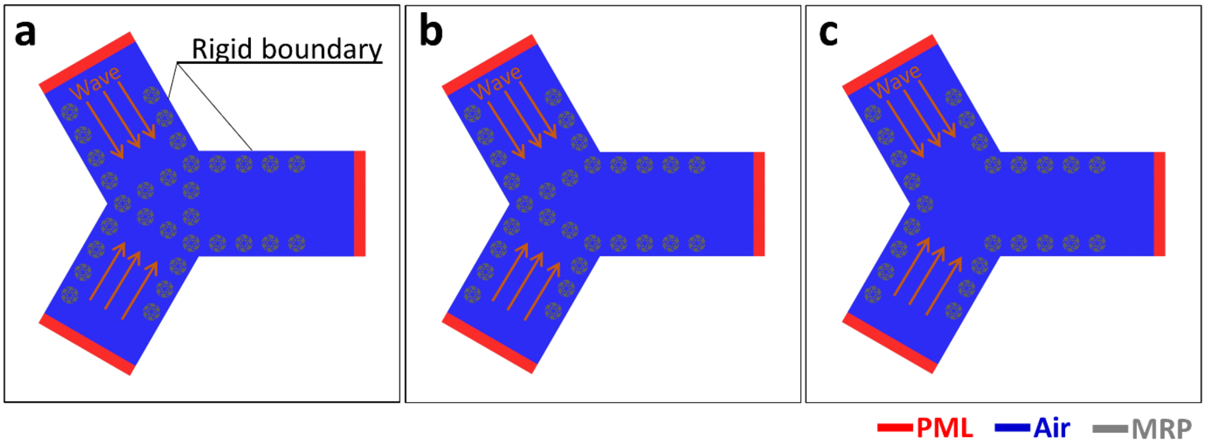

Figure S11. Configurations of the numerical simulations for the magnetoactive logic gates shown in **Fig. 4**: **(a)** NOT gate, **(b)** AND gate, and **(c)** OR gate. Note that the bent pillars in b and c are removed in the simulations.

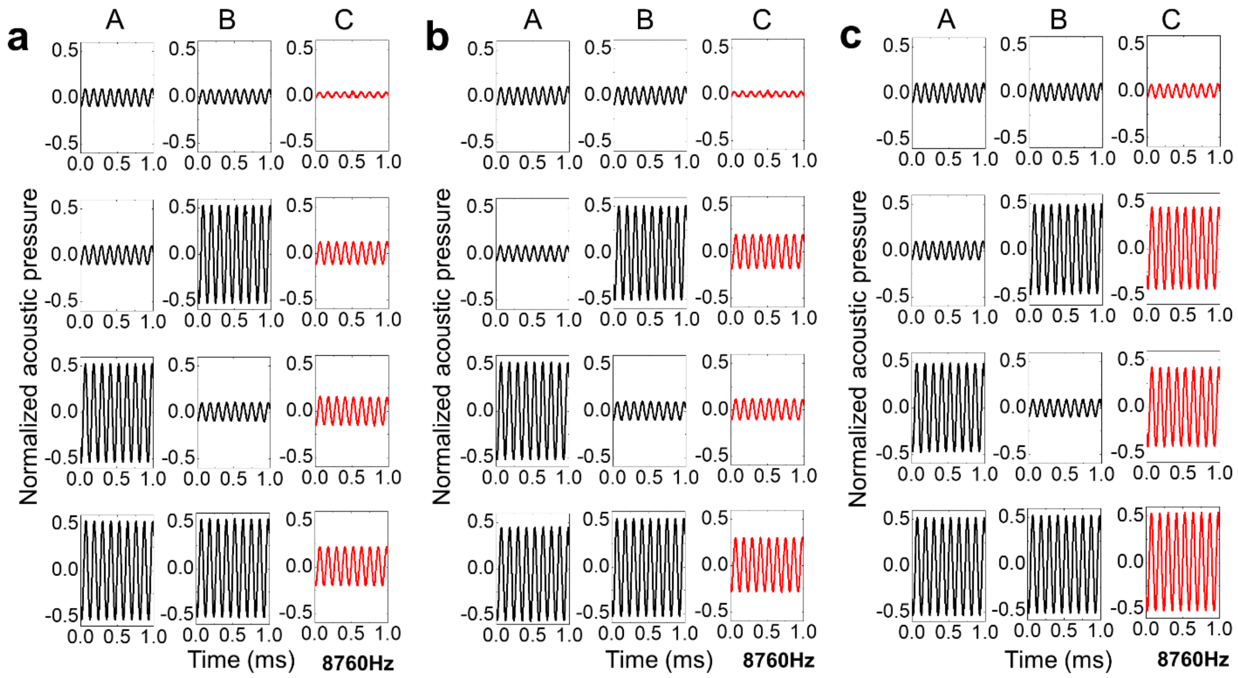

Figure S12. Experimentally measured acoustic pressure signals at 8760Hz for the magnetoactive logic gates shown in **Fig. 4**: **(a)** NOT gate, **(b)** AND gate, and **(c)** OR gate.

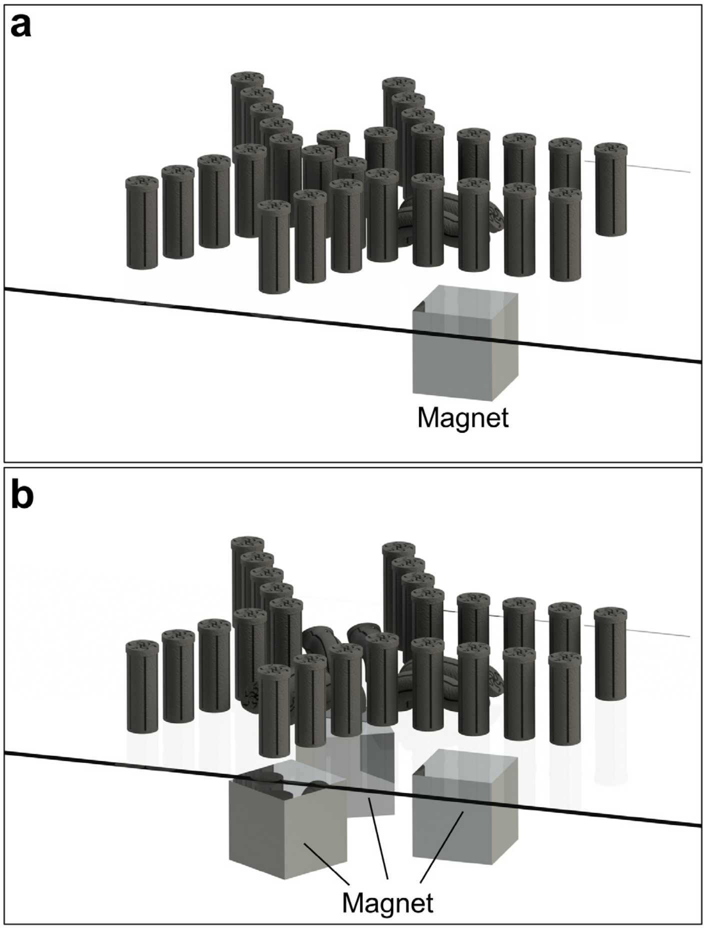

Figure S13. (a) Schematic to show the application of magnetic fields to bend 2 pillars shown in **Fig. 4g**. (b) Schematic to show the application of magnetic fields to bend 6 pillars shown in **Fig. 4l**. The selective actuation of the central six pillars is because of their higher iron volume fraction (18.48% by weight). The pillars for the channel walls have lower iron volume fraction (3.14% by weight).

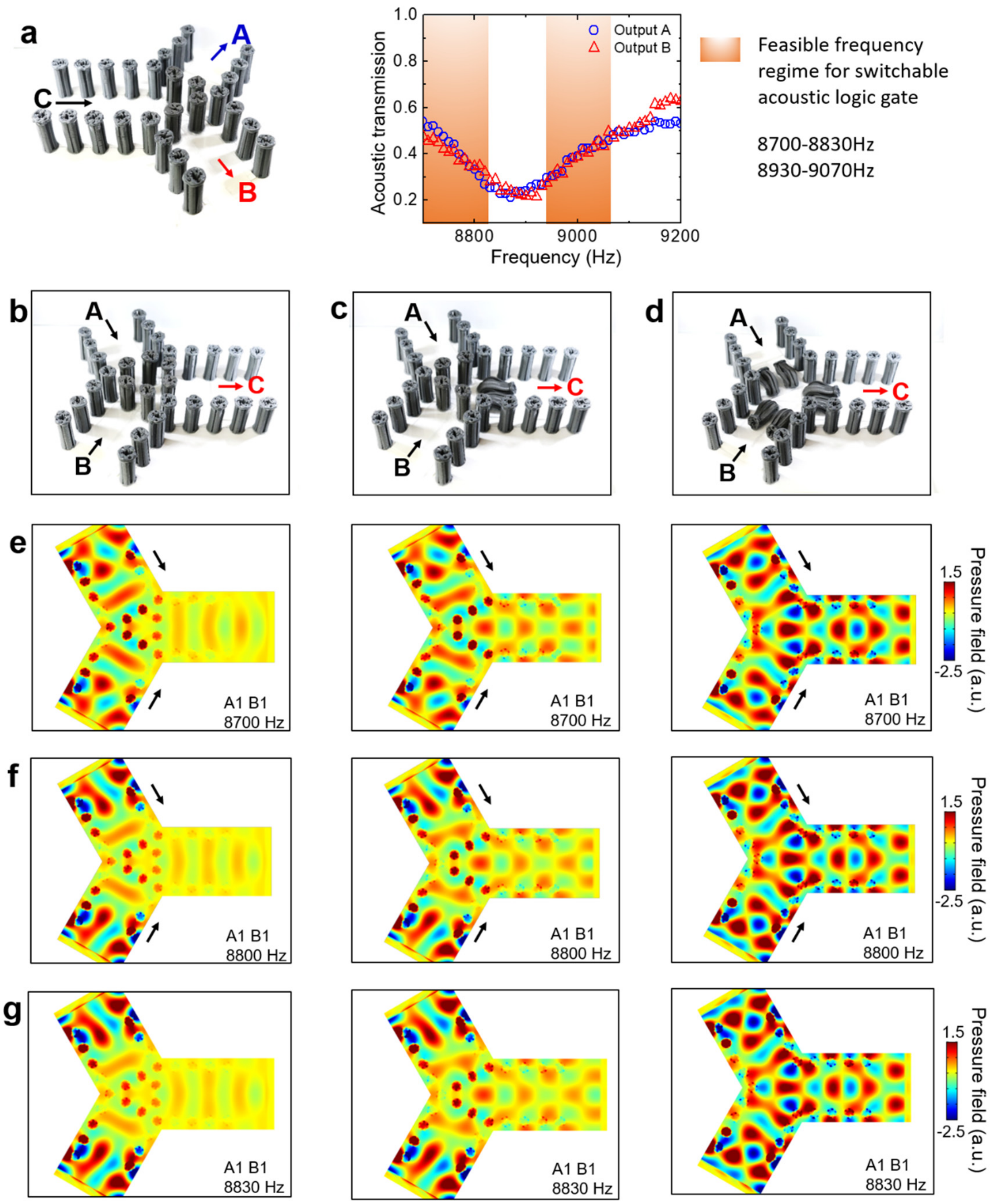

Figure S14. **(a)** Schematic and data to reveal the working frequency range for the magnetoactive reconfigurable acoustic logic gate shown in **Fig. 4**. The shadowed areas indicate the feasible working frequency: 8700-8830 Hz and 8930-9070 Hz. Schematics for NOT gate **(b)**, AND gate **(c)**, and OR gate **(d)**, and the corresponding numerical simulations at 8700 Hz **(e)**, 8800 Hz **(f)**, and 8830 Hz **(g)**.

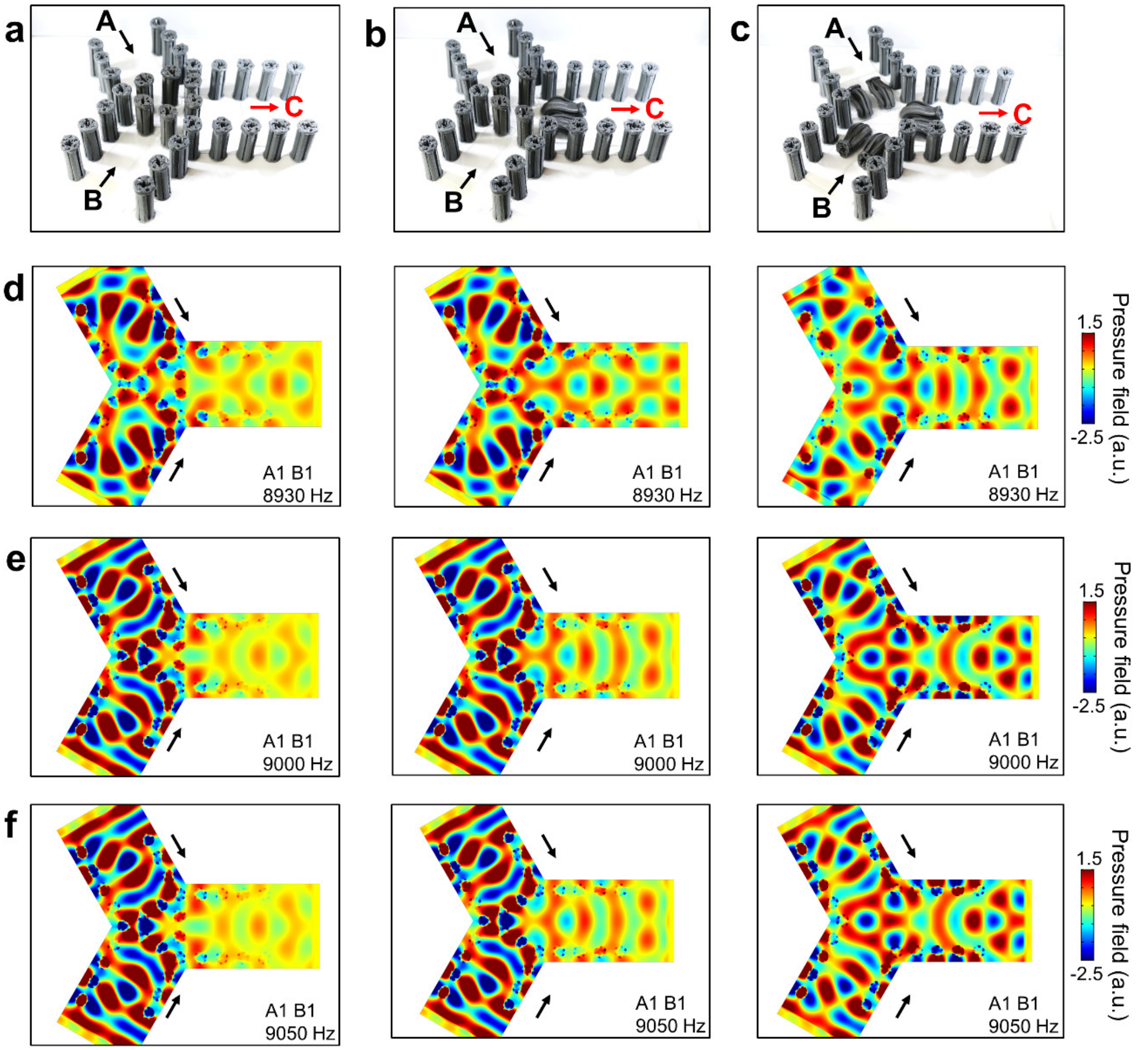

Figure S15. Schematics for NOT gate **(a)**, AND gate **(b)**, and OR gate **(c)**, and the corresponding numerical simulations at 8930 Hz **(d)**, 9000 Hz **(e)**, and 9050 Hz **(f)**.

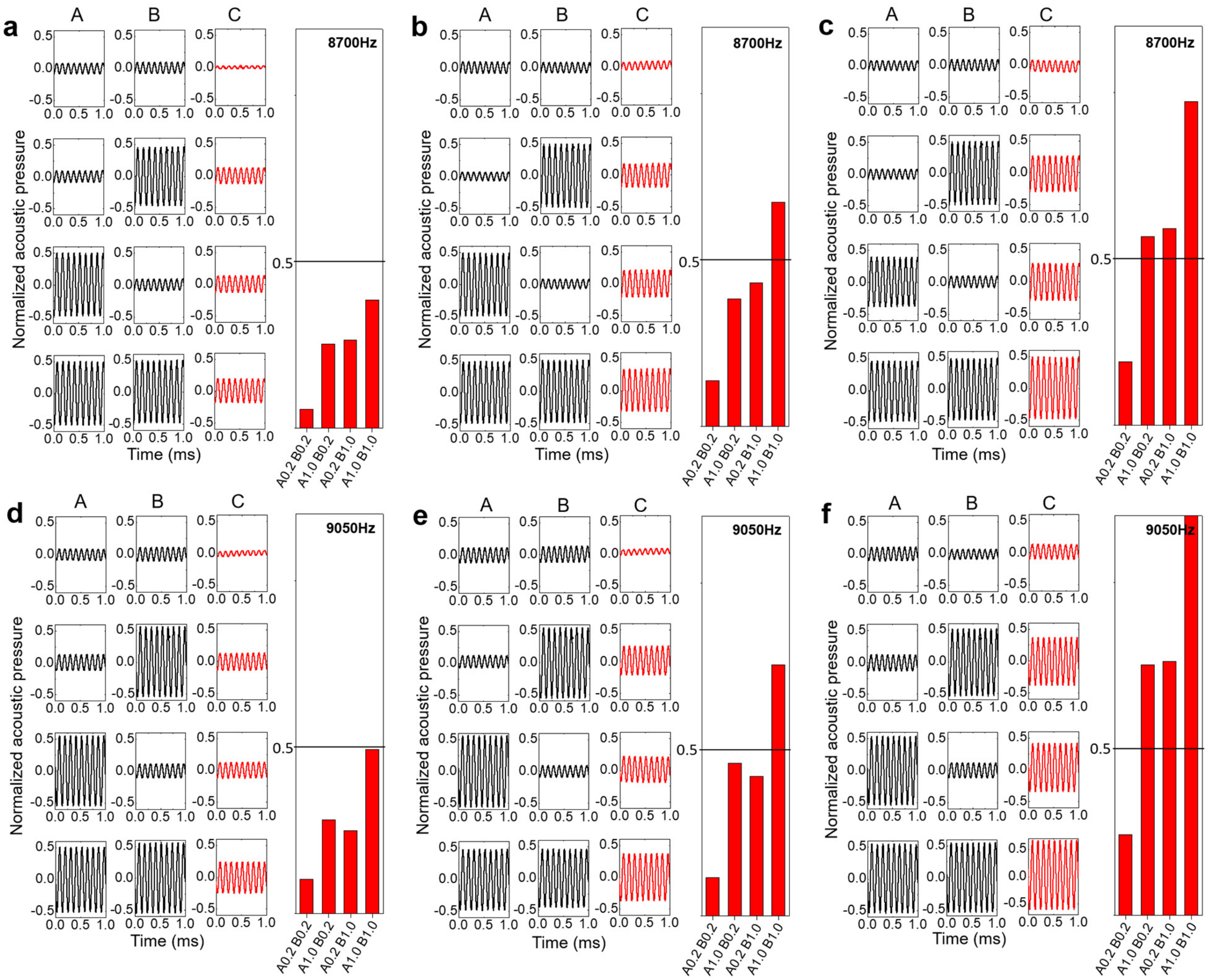

Figure S16. Experimentally measured acoustic pressure signals at **(a-c)** 8700Hz and **(d-f)** 9050Hz for the magnetoactive logic gates: **(a, d)** NOT gate, **(b, e)** AND gate, and **(c, f)** OR gate.

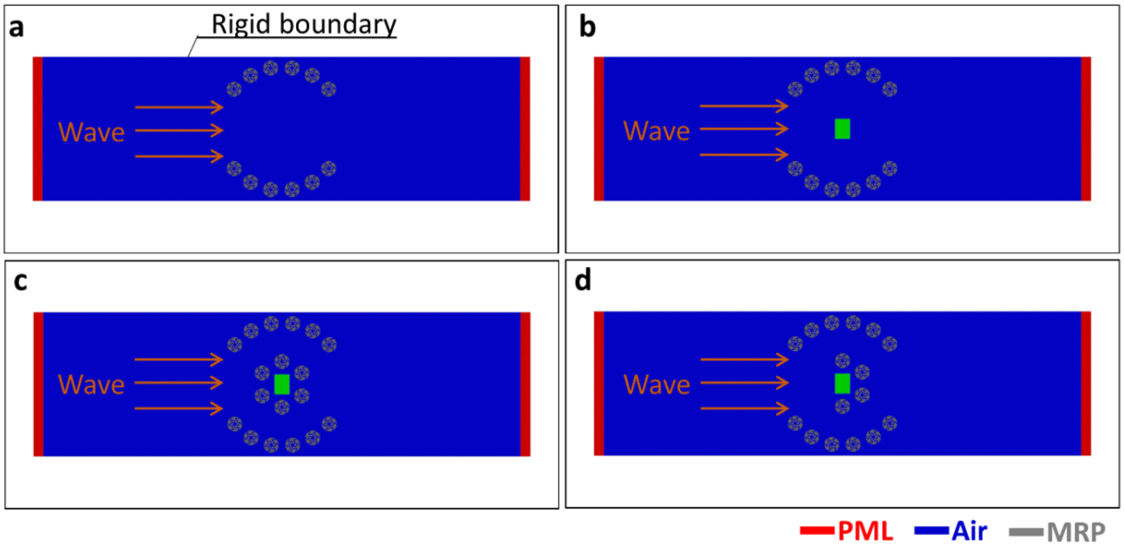

Figure S17. Configurations of the numerical simulations for the magnetoactive acoustic cloak with four states shown in **Fig. 5**: **(a)** reference, **(b)** object, **(c)** cloaked, and **(d)** uncloaked.

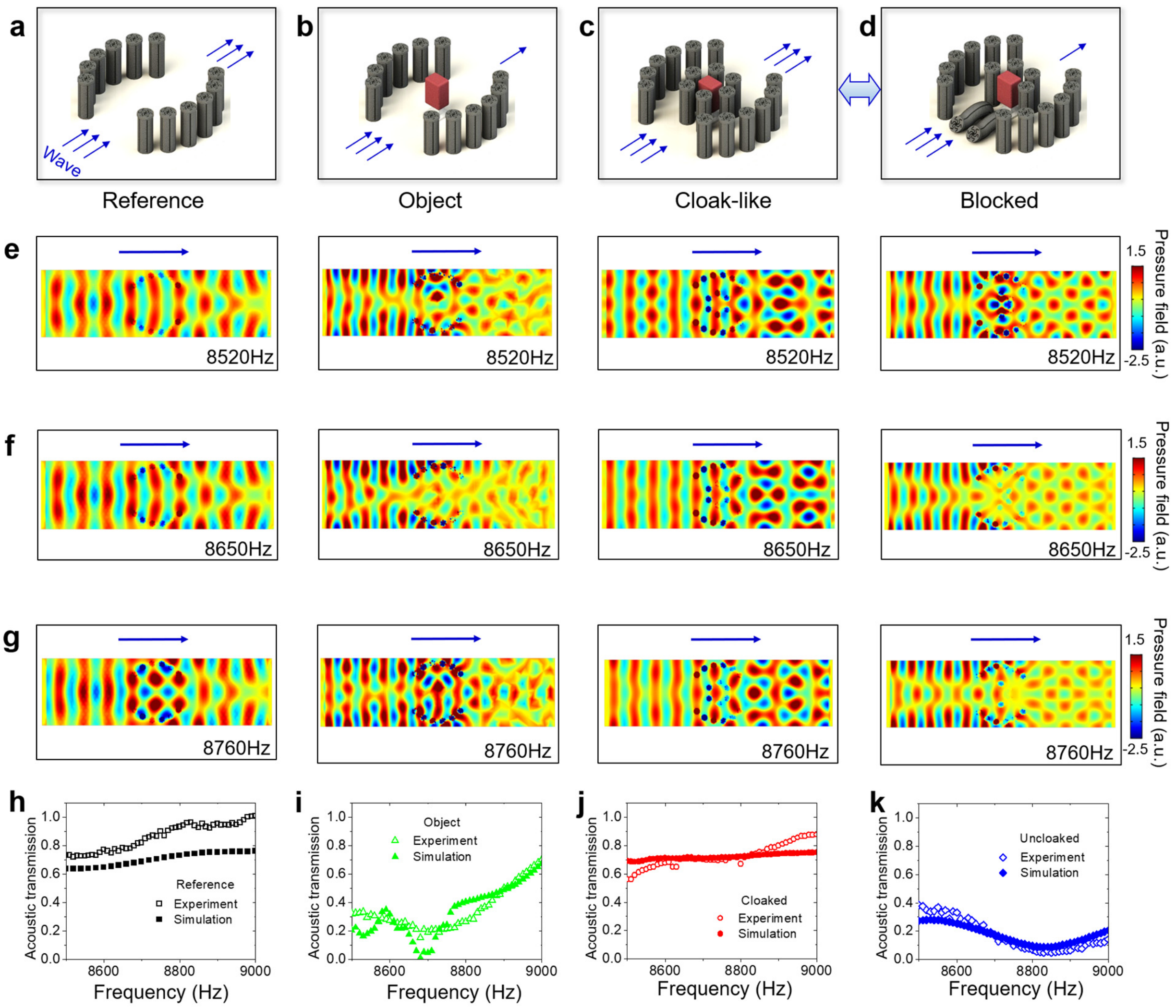

Figure S18. Schematics for reference state **(a)**, object state **(b)**, cloak-like state **(c)**, and blocked state **(d)**, and the corresponding numerical simulations at 8520 Hz **(e)**, 8650 Hz **(f)**, and 8760 Hz **(g)**. **(h-k)** The experimentally measured and numerically simulated acoustic transmission for four states in functions of the frequency.

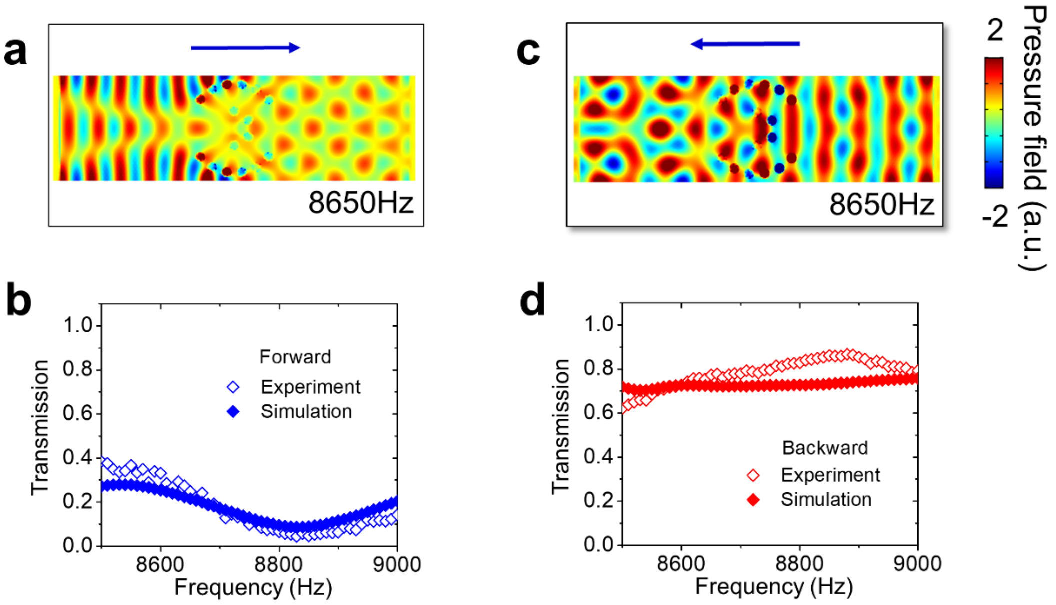

Figure S19. Numerical simulations **(a, c)** and acoustic transmissions **(b, d)** for the forward **(a, b)** and backward **(b, d)** acoustic directions.

6. Supplementary movies

Movie S1: Magnetically-induced cyclic bending of the central two pillars of a Mie resonator pillar (MRP) array. The speed of the movie is three times of the normal speed. The cycle period is around 5 s.

Movie S2: Magnetically-induced cyclic bending of the central two pillars of a Mie resonator pillar (MRP) array. The movie is real time. The cycle period is around 1 s.

Movie S3: Magnetically-induced cyclic bending of the front two pillars within an MRP-enabled cloak-like waveguide. The speed of the movie is three times of the normal speed.

Reference

1. Ball P. Engineering shark skin and other solutions. *Nature* **400**, 507 (1999).
2. Wen L, Weaver JC, Lauder GV. Biomimetic shark skin: design, fabrication and hydrodynamic function. *The Journal of Experimental Biology* **217**, 1656-1666 (2014).
3. Dean B, Bhushan B. Shark-skin surfaces for fluid-drag reduction in turbulent flow: a review. *Philosophical Transactions of the Royal Society A: Mathematical, Physical and Engineering Sciences* **368**, 4775-4806 (2010).
4. Bechert D, Bruse M, Hage Wv, Van der Hoeven JT, Hoppe G. Experiments on drag-reducing surfaces and their optimization with an adjustable geometry. *Journal of fluid mechanics* **338**, 59-87 (1997).
5. Domel AG, Saadat M, Weaver JC, Haj-Hariri H, Bertoldi K, Lauder GV. Shark skin-inspired designs that improve aerodynamic performance. *Journal of the Royal Society Interface* **15**, 20170828 (2018).
6. Oeffner J, Lauder GV. The hydrodynamic function of shark skin and two biomimetic applications. *Journal of Experimental Biology* **215**, 785-795 (2012).
7. Cheng Y, Zhou C, Yuan B, Wu D, Wei Q, Liu X. Ultra-sparse metasurface for high reflection of low-frequency sound based on artificial Mie resonances. *Nature materials* **14**, 1013 (2015).
8. Liang Z, Li J. Extreme acoustic metamaterial by coiling up space. *Physical review letters* **108**, 114301 (2012).
9. Ying C, Xiao-Jun L. Extraordinary Resonant Scattering in Imperfect Acoustic Cloak. *Chinese Physics Letters* **26**, 014301 (2009).
10. Hu X, Ho K-M, Chan CT, Zi J. Homogenization of acoustic metamaterials of Helmholtz resonators in fluid. *Physical Review B* **77**, 172301 (2008).
11. Yu K, Fang NX, Huang G, Wang Q. Magnetoactive acoustic metamaterials. *Advanced Materials* **30**, 1706348 (2018).
12. Yih-Hsing P, Chau-Shioung Y. A linear theory for soft ferromagnetic elastic solids. *International Journal of Engineering Science* **11**, 415-436 (1973).
13. Landau LD, Lífshíts EM, Pitaevskii L. *Electrodynamics of Continuous Media*. Pergamon press Oxford (1984).
14. Kankanala SV, Triantafyllidis N. On finitely strained magnetorheological elastomers. *Journal of the Mechanics and Physics of Solids* **52**, 2869-2908 (2004).
15. Moon FC, Pao Y-H. Magnetoelastic Buckling of a Thin Plate. *Journal of Applied Mechanics* **35**, 53-58 (1968).
16. Gerbal F, Wang Y, Lyonnet F, Bacri J-C, Hocquet T, Devaud M. A refined theory of magnetoelastic buckling matches experiments with ferromagnetic and superparamagnetic rods. *Proceedings of the National Academy of Sciences* **112**, 7135-7140 (2015).
